# Mechanics of Epoxy Nanocomposites: A Study on the Synergy of the Reinforcements

**DOI:** 10.1021/acsomega.5c10757

**Published:** 2026-02-18

**Authors:** İnci Pir, Mertol Tüfekci, Seren Acarer Arat, Ekrem Tüfekci

**Affiliations:** † Istanbul Technical University, Faculty of Mechanical Engineering, Gumussuyu Istanbul 34467, Turkey; ‡ Centre for Engineering Research, University of Hertfordshire, College Lane Campus, Hatfield AL10 9AB, U.K.; § School of Physics, Engineering and Computer Science, University of Hertfordshire, College Lane Campus Hatfield AL10 9AB, U.K.; ∥ 52971 Istanbul Technical University, Department of Environmental Engineering, MasRlak, Istanbul 34437, Turkey

## Abstract

In this study, manufacturing and mechanical characterization are performed on halloysite nanotube (HNT)-reinforced epoxy composite, carboxyl-terminated butadiene-acrylonitrile (CTBN) rubber-added epoxy composite, and both HNT- and CTBN-rubber-added epoxy composites. It is aimed to explore the effects of HNT and CTBN rubber inclusions individually and the synergistic effects of HNT and CTBN rubber inclusions on the epoxy-based composite material. To achieve this, the mechanical characterization of the epoxy matrix composite is performed numerically and experimentally. To investigate the viscoelastic behavior, the samples are subjected to tensile and three-point bending tests at different strain rates (%1, %5, and %10 strain per minute) and to Charpy impact tests. The internal structures of the samples are observed using a scanning electron microscope (SEM). Results demonstrate that 1% HNT reinforcement increases the elastic modulus by 15% (from 599 to 688 MPa in tensile tests), while 10% CTBN rubber reduces stiffness by 38% but increases elongation at break by 48%. Hybrid composites (H10R05) achieve balanced properties with 16% higher stiffness than pure rubber systems while maintaining 44% higher ductility than pure epoxy. Charpy impact tests show that 10% rubber increases fracture energy by approximately 85% compared to pure epoxy, while HNT provides modest improvements. All samples exhibit strain-rate-dependent behavior, with elastic modulus increasing 10–16% from quasi-static to dynamic loading rates. Numerical modeling using the Mori–Tanaka, Halpin–Tsai, and finite element homogenization (FEH) methods successfully predicts experimental trends, with FEH showing the highest accuracy (deviations <5%). This study provides valuable insights into designing composite materials with balanced mechanical properties through multireinforcement strategies.

## Graphical abstract

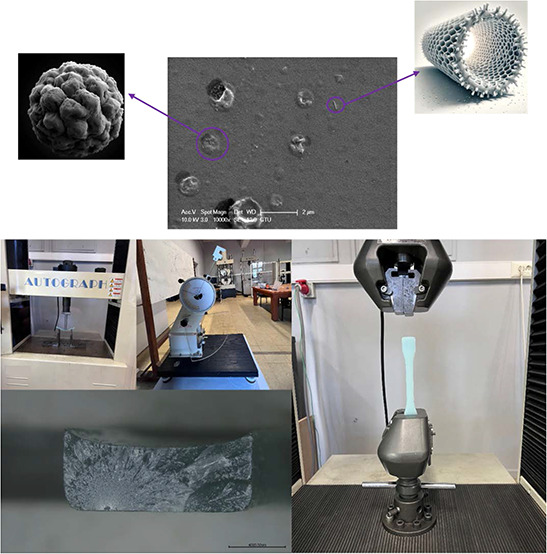

## 1 Introduction

In polymer matrix composites, high-strength reinforcements are held together by a polymer matrix. Reinforcement materials are designed to support the mechanical loads to which the composite structure is exposed.^1^ Polymer matrix composites have broader use areas than other composites due to their ease of manufacture, low cost, and improved mechanical properties. Wind turbine blades can be given as an example of the use of composite materials in the energy field.^2^

The dispersion of nanometer-sized fibers/inclusions in the matrix forms nanocomposites. Material properties at the nanoscale can be considerably different compared to the material properties of the same material on the macroscale. As the dimensions of the inclusions get smaller, their surface-to-volume ratio increases, so, the interfaces for bonding with the matrix become much more influential on the mechanical properties as the material defects decrease. Fattah et al., examining the chemical interaction of different sizes of fumed silica particles with epoxy, state that more efficient results are obtained from small-sized particles.^3^ Experimental results show that mechanical properties, such as the modulus of elasticity, are size-dependent.^4–6^

Epoxy resins are commonly chosen as a matrix material in composites.^7^ Furthermore, they serve as a structural matrix in high-performance polymer composites.^8^ In epoxy matrix composites, the matrix is mechanically strengthened with various reinforcements with resilient mechanical properties. Comprehensive studies on epoxy nanocomposites using nanosized additives such as nanoparticles (i.e., fumed silica, TiO_2_, and Al_2_O_3_), nanotubes (i.e., carbon nanotube (CNT), halloysite nanotube (HNT)), fibers, and nano clays are found in the literature due to their resilient mechanical properties in improving the mechanical performance of epoxy composites.^9–14^

HNT is used to improve the mechanical and thermal properties of polymeric nanocomposites.^15^ Due to its geometry/structure, HNT reinforcement helps to strengthen the composite material by carrying more load, causing a load transfer from the matrix to the nanotubes.^16^ Besides, a stiffer behavior can be seen in the HNT-reinforced epoxy composites,.^11,17^ Ravichandran et al. investigated the mechanical properties of HNT-doped epoxy nanocomposites. They found that the mechanical properties of HNT-doped nanocomposites are improved compared to pure epoxy since HNT restricts the deformation and movement of the epoxy matrix.^17^ Recent investigations have demonstrated enhanced performance through HNT functionalization. Studies have shown that functionalized HNTs can simultaneously improve both flame retardancy and mechanical properties in epoxy systems.^18^

With rubber inclusions, increases in the fracture energy, fracture toughness, and impact resistance of the composite material can be expected. Rubber addition to the epoxy matrix is discussed in various ways in the literature.^19^ In some studies, rubber inclusions increase the measured toughness value of the composite material.^20–22^ In other studies, it is observed that adding rubber inclusions to the matrix increases the damping properties of the composite material.^23–25^ Some studies show that adding carboxyl-terminated butadiene-acrylonitrile (CTBN) rubber to epoxy resin reduces the stiffness properties of composites,.^26,27^ Xu et al. showed that the elastic modulus and yield stress values decrease significantly after adding CTBN rubber to the epoxy resin. Hybrid composites reinforced with nano silica and CTBN rubber particles are also studied in order to obtain optimum strength and toughness levels in epoxy resin,.^14,28^ Mansour et al.^29^ experimentally examined epoxy composites in which CTBN rubber was mixed in different ratios. In their research, it was observed that the stiffness of the epoxy-CTBN rubber composites decreased significantly. The lowest elasticity modulus of the composite material is measured when 25% by weight CTBN rubber is added. It is determined that the measured elasticity modulus decreased by 56% compared to that of pure epoxy.^29^ Contemporary studies continue to advance rubber-toughening strategies. A comprehensive review highlighted the evolution of hybrid approaches.^30^ Additionally, rubber-composite-nanoparticle modifications can achieve both low curing temperatures and high toughness.^31^ Hybrid epoxy nanocomposites require understanding toughening mechanisms for material design.^32^

There are various studies on the determination of the material properties of composite materials.^33–36^ Tensile test, three-point bending test, Charpy impact test, and dynamic mechanical analysis (DMA) can be examples of standardized experimental methods in the mechanical characterization of composite materials,.^11,37,38^ Apart from standard methods, there are methods that are developed and proposed to test composite materials, aiming to measure properties like damping.^39–41^

Even though tensile tests and three-point bending tests are usually employed to determine the mechanics under quasi-static loading/deformation conditions, it is worth noting that they can also be utilized to characterize the mechanical properties of materials in which the loading speed has an impact on the results of the measurements and, consequently, the determined mechanical properties. The influence of strain rate on nanocomposite fracture behavior has gained significant attention. Multiscale studies on strain rate effects in polymer nanocomposites provide insights that align with the rate-dependent characterization approach employed in this work.^42^ Viscoelastic materials, which can be defined as materials whose behavior depends on the loading rate, are among such materials.^43^ For these materials, low-speed tests may reveal low strength and high ductility behavior, whereas high-speed tests may exhibit high strength and low ductility characteristics.

The Charpy impact test determines the fracture energy of the material. This test provides information about the ductile or brittle behavior of the material by measuring the energy absorbed during a sudden impact. A key aspect of this test involves accurately measuring the point at which the sample breaks under a dynamic load. In the literature, this method is used to compare the fracture behavior of materials.^44–46^

Aside from experimental methods, mathematical models are also used in composite material modeling. Applying these techniques saves time and cost, since they are well-known and validated, which can be used as a basis for design. Homogenization methods are among those mathematical modeling techniques that can be used to model the mechanical behavior of many engineering materials.^47,48^ Recent theoretical developments have extended the Mori–Tanaka framework, presenting advanced formulations for general interfaces and enhancing the method’s applicability to complex nanocomposite systems.^49^

Based on the literature, it is found that neither HNT nor rubber independently provides both rigid and ductile behavior in a structure. When rubber inclusions are added o epoxy, the material exhibits more ductile behavior, but at the expense of decreased rigidity. Conversely, the reinforcement of HNT in the epoxy matrix results in more rigid behavior.

This study presents a comprehensive experimental and numerical characterization of epoxy-based nanocomposites reinforced with HNT, CTBN rubber, and their hybrid combination. The strain-rate-sensitive mechanical properties of these composite samples are experimentally assessed through tensile and three-point bending tests at three different strain rates. Besides, Charpy impact tests are performed to examine the fracture energies and ductility of the epoxy-based composites. Scanning electron microscopy (SEM) and stereomicroscopy are conducted to verify the structural integrity. Numerical simulations, utilizing the Mori–Tanaka homogenization method, the Halpin–Tsai model, and the finite element homogenization approach, are employed to validate the experimental findings. The primary novelty of this study lies in the systematic experimental and numerical investigation of synergistic effects when HNT (stiffening) and CTBN rubber (toughening) reinforcements are combined. While individual effects are documented in the literature, the comprehensive characterization of their combined behavior across varying strain rates and their validation through multiple numerical approaches remains limited. This work demonstrates that predictable intermediate mechanical properties can be achieved, enabling a tailored stiffness–toughness balance for specific applications.

## 2 Material Manufacturing and Characterization Methods

This section presents the sample preparation and the experimental techniques used to characterize the manufactured nanocomposites. The characterization consists of mechanical tests, including tensile, three-point bending, and Charpy impact tests. To visualize the internal structure of the prepared samples, SEM is also performed.

### 2.1 Material Preparation and Manufacturing Process

To investigate the synergistic effects of HNT and rubber in epoxy matrix composites and the impact of material composition on the mechanical properties, samples are manufactured using a consistent, standardized manufacturing procedure. The MGS L 285 epoxy resin and the MGS H 287 hardener set by Hexion are chosen as the matrix. The HNT used in this study is a nanotube, with the diameter of the reinforcement material varying in the range of 30–70 nm and the length ranging from 1 to 3 μm. The HNT reinforcement is acquired from Nanografi Nanotechnology. From the literature, the CTBN reinforcement sizes are reported based on SEM images, and they are spherical particles with diameters in the range of 0.5–1 μm.^50^ For CTBN rubber-reinforced composites, the Albipox1000 is used, which is a commercial product that contains CTBN rubber m_f_ = 40%, provided by Evonik Industries, AG, Germany. The CTBN rubber in Albipox 1000 is carboxyl-terminated, containing reactive carboxyl functional groups (−COOH) at the polymer chain ends. During the curing process, these terminal groups chemically react with epoxy groups, forming covalent bonds at the rubber–epoxy interface. This chemical bonding mechanism ensures strong interfacial adhesion, enabling effective stress transfer between the rubber phase and the epoxy matrix, which is critical for the toughening effect. Table 1 presents the physical and mechanical properties of the epoxy resin and reinforcement material. Figure 1 presents the image of the HNT and Albipox 1000 reinforced with CTBN rubber.

**Table 1:** Properties of the Constituent Materials

Material	Property	Value
Epoxy	Density	1.15 g/cm^3^
	Poisson’s Ratio	0.35
	Tensile Modulus	598.9 MPa*
	Flexural Modulus	2410.8 MPa*
* Measured in this study at quasi-static strain rate (0.01 strain/min)		
HNT	Density	2.53 g/cm^3^
	Diameter	30–70 nm
	Length	1–3 μm
CTBN Rubber	Density	∼0.98 g/cm^3^
	Diameter	0.5–1 μm
	Length	0.5–1 μm
	Mass Fraction in Product	40%

**Figure 1:**
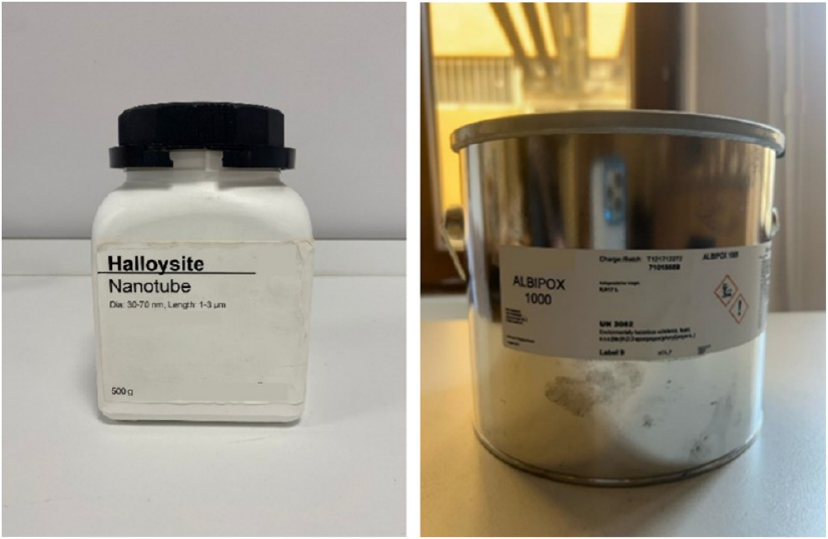
Pictures of the HNT and Albipox 1000.

For the composites that contain only HNT, the reinforcements are added to the resin at 0.5% and 1% in the mass fraction (m_f_). For CTBN rubber-reinforced epoxy samples, the reinforcements are mixed into the epoxy resin at 5% and 10% (m_f_). To investigate the synergistic effects, 5% CTBN rubber and 0.5% HNT, 5% CTBN rubber 1% HNT, 10% CTBN rubber and 0.5% HNT, and 10% CTBN rubber 1% HNT by weight in the epoxy matrix composites are prepared. For easy identification, specimen codes are defined, and the detailed compositions (mass fractions) of the samples are given in Table 2.

**Table 2:** Composition of Manufactured Samples, with Respect to the Reinforcement Type

Manufactured Sample Type	m_f_ of HNT (%)	m_f_ of CTBN Rubber (%)
Pure Epoxy Sample—PE	0	0
0.5% HNT Reinforced Sample—H05	0.5	0
1% HNT Reinforced Sample—H1	1	0
5% Rubber Reinforced Sample—R5	0	5
10% Rubber Reinforced Sample—R10	0	10
0.5% HNT and 5% Rubber—H05-R5	0.5	5
1% HNT and 5% Rubber—H1-R5	1	5
0.5% HNT and 10% Rubber -H05R10	0.5	10
1% HNT and 10% Rubber—H1R10	1	10

In order to reduce and minimize the captured humidity between the HNT particles, HNT is kept in an incubator at 60̊C for 6 h before starting the manufacturing process. The reinforcement and epoxy are mixed in a magnetic stirrer for about 60–90 min until homogenization is achieved. The combination of HNT and epoxy is intermittently mixed with an ultrasonic homogenizer at 30% amplitude frequency for 5 min. During this process, the mixture’s temperature is controlled to prevent any chemical reactions caused by excessive heating, which may strongly affect the mechanical properties of the epoxy resin. This multistep dispersion process, combining mechanical stirring, ultrasonication, and controlled thermal processing, has been demonstrated to achieve effective nanoparticle distribution in epoxy matrices. The intermittent ultrasonication approach prevents excessive heating while providing sufficient energy for particle deagglomeration. Temperature monitoring during processing ensures that the epoxy remains below its curing temperature, preserving the resin’s mechanical properties. After the ultrasonic homogenizing, a vacuum is applied to the mixtures for 2 h. Following the mixing and vacuuming process, the mixture is poured into a Teflon mold for casting. The mold is placed in the vacuum chamber, and the mixture is cured at room temperature and vacuumed for 24 h. Then, samples are kept in an incubator at 60 °C for 15 h postcuring.

### 2.2 Experimental Characterization Procedures

Tensile, three-point bending, Charpy impact tests, and SEM are employed to characterize the manufactured samples. All mechanical experiments are repeated until three consistent data sets are obtained for each material. To better understand and evaluate the strain rate sensitivity of the mechanical properties exhibited by the respective materials, tensile and three-point bending tests are conducted on HNT-reinforced epoxy nanocomposites, CTBN rubber-reinforced epoxy nanocomposites, composites containing both HNT and CTBN rubber reinforcements, and unreinforced epoxy. These assessments are performed under a range of strain rates to elucidate the relationship between strain rate and the mechanical behavior of each type of nanocomposite. Three strain rates (0.01, 0.05, and 0.1 strain/min) are selected to show the transition from quasi-static to dynamic loading conditions. The lowest rate, 0.01 strain/min, corresponds to quasi-static loading and aligns with standard ASTM testing protocols, providing a baseline for comparison. The highest rate, 0.1 strain/min, represents a 10-fold increase in loading speed, enabling the assessment of rate-dependent viscoelastic behavior without exceeding the operational limits of the universal testing machine. This order-of-magnitude range is sufficient to capture the strain-rate sensitivity of epoxy-based composite materials, which exhibit pronounced viscoelastic effects under varying loading rates. Furthermore, these parameters reflect practical engineering scenarios, ranging from slow assembly stresses to moderate dynamic service loads, ensuring that the study remains both experimentally feasible and structurally relevant. Besides, Charpy impact tests are performed to gain an understanding of the impact performance of the composite samples. As the final part of the experimental characterization, representative images of the broken cross sections are acquired using SEM, aiming to visually confirm the absence of significant material flaws.

#### 2.2.1 Tensile Tests

Tensile tests are conducted to gain insights into the composite material’s performance at distinct strain rates. In terms of mechanical properties, this research focuses on the elasticity modulus, tensile strength, and elongation at break. The tests are executed on the Shimadzu AG-IS 50 kN Universal Testing Apparatus at three disparate strain rates: 0.01 (also referred to as quasi-static), 0.05, and 0.1 strain per minute. Tensile test specimens are prepared according to ASTM D638 Type I geometry, with an overall length of 165 mm, a narrow-section width of 13 mm, a gauge length of 50 mm, and a thickness of 4 mm.^51^ A visual representation of both the sample and test setup is given in Figure 2.

**Figure 2:**
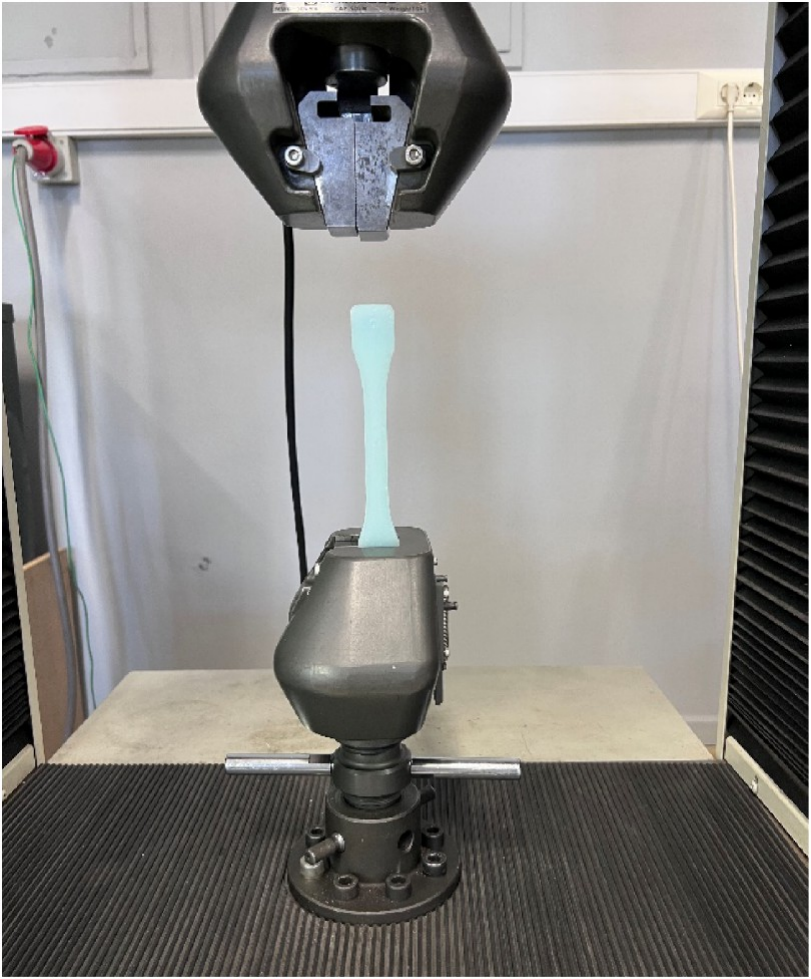
Tensile testing sample and test setup.

#### 2.2.2 Three-Point Bending Tests

Three-point bending tests are conducted to obtain information about the behavior of the composite material under bending load at varying strain rates, which correspond to the same values as the ones used for the tensile tests. Three-point bending tests are also performed on the Shimadzu AG-IS 50 kN Universal Test Machine. Three-point bending specimens are prepared according to ASTM D790 specifications, with a length of 128 mm and a width of 13 mm.^52^ An image of the threepoint bending test installation is given in Figure 3.

**Figure 3:**
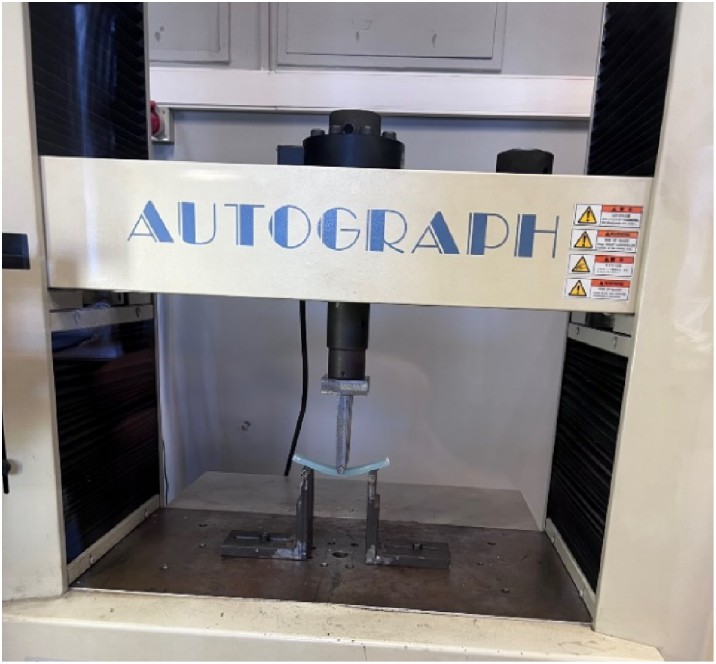
Three-point bending test setup.

#### 2.2.3 Charpy Impact Tests

After characterizing the mechanical properties of nanocomposites depending on the strain rate, Charpy impact tests are carried out to compare the fracture energies of the manufactured samples with the Devotrans DVT CD48 Test Machine. These experiments aim to reveal the effect of reinforcement type and mass fraction on the fracture energy of the epoxy matrix. Charpy impact test specimens are prepared following the ASTM D6110 standard, with a 127 mm length, 12.7 mm width, and V-notches machined to ∼2 mm depth.^53^ The tests are repeated six times for each sample. The Charpy impact test setup is visualized in Figure 4.

**Figure 4:**
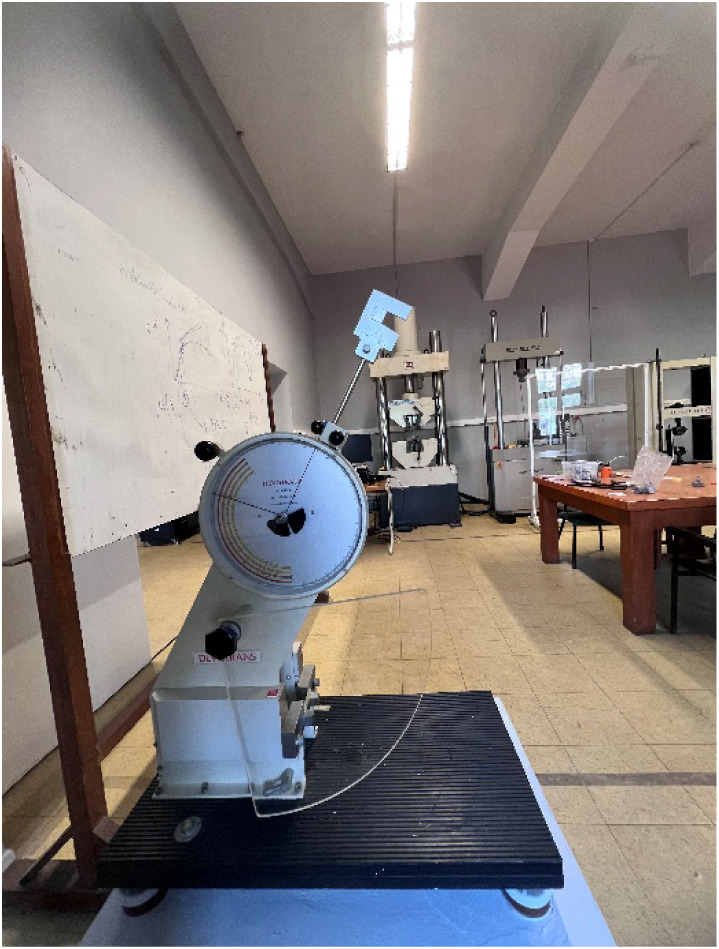
Charpy impact test setup.

#### 2.2.4 Scanning Electron Microscopy

SEM determines the physical properties of nanoscale materials. The purpose of taking SEM images is to monitor the dispersion of inclusions in the nanocomposites. Furthermore, critical flaws in the composite material that may lead to a notch effect can also be determined with SEM,.^54,55^ In SEM imaging, scanning occurs within a vacuum with electrons directed toward the surface and subsequently reflected. The surface images of the samples are examined using a scanning electron microscope (Philips XL 30S FEG). The samples are coated with gold using a coating device (Quorum SC7620) to make them conductive. SEM surface images of the samples are obtained at 10000× magnification and 10 kV accelerating voltage.

## 3 Numerical Modeling Methods

Following the experimental characterization, numerical modeling of the composites is carried out. To gain a further understanding of the influence of the nanosized inclusions on the mechanical properties of the composites, two separate mathematical modeling approaches are employed. The Mori–Tanaka homogenization method and the Halpin–Tsai model are frequently employed for modeling nanoparticle-reinforced composites in the literature, as evidenced by numerous studies.^56–59^

### 3.1 Mori–Tanaka Mean-Field Homogenization Method

The Mori–Tanaka homogenization method, employing closed-form and analytical equations to deduce composite materials’ properties, is extensively utilized for particle-reinforced nanocomposites due to its mathematical aspects and applicability, as evidenced in numerous studies.^60–66^

Homogenization methods used in material modeling studies can be evaluated by commercial software. The Mori–Tanaka homogenization method is one of these examples, and Digimat MF software from MSC Software is one of the examples of this software. Digimat MF software is a multiscale material modeling software based on the Eshelby solution and the Mori–Tanaka model for micromechanical analysis. The geometric properties, distribution, and location of reinforcement in the composite material are introduced as input to this program. The elasticity modulus and aspect ratio values of the reinforcement and matrix are used to define the material properties of the simulated composite layers. The first-order homogenization technique used is carried out in three steps:

1. The strain tensor is calculated for each integration point of the macroscopic mesh.
2. The strain tensor of a macroscopic point is used to formulate the boundary conditions applied to the representative volume element. These conditions cause the representative volume element to change shape.
3. The stress tensor of the initial macroscopic point is calculated by averaging the stress field in the representative volume element over the volume of the representative volume element.

### 3.2 Halpin–Tsai Model

Another method to determine the mechanical properties of composite materials is the Halpin–Tsai model, which was developed through curve fitting based on elasticity theory by Halpin and Tsai.^67^ This method is a homogenization model that takes into account the elastic properties of the matrix and reinforcement materials and their proportions within the composite structure. Numerous studies in the literature utilize the Halpin–Tsai model for polymer matrix nanocomposites,^68^−,^69,70^ with the composite material’s elasticity modulus determined via equations provided by Yeh et al.^69^ In this study, numerical analysis and modeling studies are carried out for the manufactured nanocomposites within the scope of this study using the Halpin–Tsai model and the Mori–Tanaka homogenization method.

### 3.3 Finite Element Homogenization Approach

The finite element homogenization (FEH) approach is another numerical modeling study that can be used to model the mechanical behavior of composite materials. With the finite element approach, the representative volume element (RVE), which is the smallest volume element that represents all properties of the composite material, and periodic boundary conditions (PBC), which indicate the periodicity of the boundary conditions of the RVE, are utilized. Selecting the appropriate RVE parameters and reinforcement volume fractions is essential. Following established guidelines, the RVEs are modeled as cubic unit cells with an edge length at least five times the largest reinforcement dimension (specifically, the HNT length of 3 μm).^71–74^ This configuration ensures representative behavior and minimizes boundary effects. This approach is effective for cases with low mass fractions and lower aspect ratios; otherwise, the RVE generation mechanism may not succeed. Compared with Mori–Tanaka and Halphin–Tsai methods, the FEH approach usually gives more accurate results. However, with the finite element approach, higher computational costs are shown.

In the FEH approach, as the first step, a finite element model of an RVE is prepared, featuring reinforcement particles with random orientation and position. Then, the nanoparticles that make up the composite material are arranged such that none intersect with each other or the boundaries of the RVE. After that, the nanocomposite models are created using the RVE with PBC under uniaxial tensile strain of 0.01, applied to one surface in small-scale simulations. As a result, the effective mechanical properties and stress distributions of the composite material can be obtained as output data. To ensure reliable and repeatable results, the analyses are performed five times for each material set.^71,73,75^ Figure 5 presents an image of an RVE sample generated for this study. In order to show the multireinforcement case, the RVE with both rubber and HNT reinforcement is presented in Figure 5a, where blue reinforcements represent rubber particles and red nanotubular structures represent HNT reinforcements. In Figure 5b, the HNT-reinforced RVE is given.

**Figure 5:**
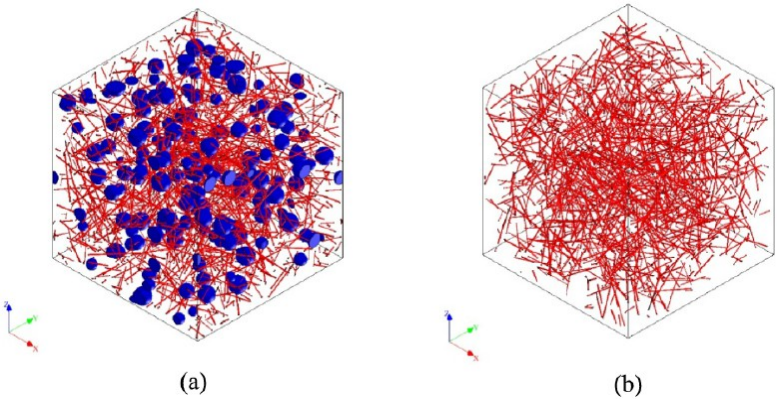
RVE images of a) Rubber and HNT-reinforced composite and b) HNT-reinforced composite studies.

## 4 Results and Discussion

In this section, the results of experimental and numerical studies conducted on the manufactured samples are presented. First, the results from the tensile test, three-point bending test, and Charpy impact test are discussed, providing critical insights into the mechanical properties of the samples with the help of experiments and a comparative examination of the fracture surfaces with a stereo microscope. Subsequently, a detailed analysis using SEM images is presented to investigate the internal structure of each sample combination, providing a microscopic perspective of the mechanical properties. Finally, the section concludes with a discussion of the numerical investigation results, integrating these findings with the experimental data to provide a comprehensive understanding of the material behavior under various testing conditions.

### 4.1 Experimental Behavior Assessment of Nanocomposites

The results of the tensile, three-point bending, and Charpy impact tests conducted to evaluate the mechanical properties of the samples at different strain rates are discussed in this section. The tensile tests and three-point bending tests are performed using three distinct strain rates: 1% strain per minute, 5% strain per minute, and 10% strain per minute. For the tensile tests, samples are subjected to uniaxial tension until failure, with controlled strain rates ensuring consistent deformation. The three-point bending tests involve applying a load at the midpoint of the sample while it is supported at two points, with the same strain rates used to observe the bending behavior and fracture characteristics. Additionally, the Charpy impact tests are conducted to assess the material’s resistance to sudden loading. The impact of these varying strain rates on the material’s mechanical performance is analyzed, providing a detailed understanding of its response under different loading conditions.

#### 4.1.1 Tensile Behavior of Nanocomposites

The elasticity modulus (E), tensile strength (σ_t_), and elongation at break (ε_b_) values obtained from tensile tests at different strain rates, and different types and amounts of reinforcements, are presented in this section.

Upon examining the results, it is observed that the stiffness of all samples exhibits consistent behavior relative to the varying strain rate, and the elasticity modulus values tend to increase with increasing strain rate. This is an expected outcome from the epoxy matrix with viscoelastic behavior.

Figures 6, 7 and Table 3 illustrate the variations in the mechanical properties of HNT-reinforced epoxy nanocomposite samples in relation to the strain rate of the tensile test and the mass fraction of HNT, in other words, reinforcement content (RC). In Figure 6, the variation of the mechanical properties with respect to the reinforcement mass fraction is visualized. In Figure 6, the change in measured values with respect to the change in mass fractions and strain rates is presented in three dimensions. In Figure 7, stress–strain diagrams with respect to the strain rate are given for each reinforcement combination. In Table 3, the obtained results are shown in a tabular format. The data indicate that the stiffness and tensile strength of the composites experience enhancement as HNT reinforcement increases, aligning with the observations corresponding to the rising strain rate. Moreover, as the HNT mass fraction within the composite structure rises, elongation at break values exhibit an increase for the quasi-static case and a decrease in other instances. These findings suggest that the material exhibits augmented ductility and toughness at lower strain rates, while it becomes increasingly brittle and loses toughness at higher strain rates. The complexity of the relationship between strain rate, reinforcement content, and material properties in HNT-reinforced epoxy nanocomposites is underscored by the trends observed in the data. It is very well-known that the material’s damping properties are subject to variation depending on the strain rate, as the theory of viscoelasticity states.

**Table 3:** Experimentally Characterized Mechanical Properties of Epoxy Reinforced with HNT in the Tensile Test

RC	EP	H05	H1
Elasticity Modulus (E) [MPa]			
Static	598.9 ± 30.7	674.4 ± 26.2	687.7 ± 36.1
5%	635.6 ± 17.5	690.5 ± 25.2	725.5 ± 58.6
10%	693.7 ± 46.2	724.5 ± 32.6	801.7 ± 38.6
Tensile Strength (σ_t_) [MPa]			
Static	60.2 ± 6.7	77.6 ± 11.9	78.6 ± 8.0
5%	68.1 ± 4.6	87.0 ± 9.1	84.5 ± 6.9
10%	74.2 ± 5.6	68.5 ± 4.9	80.1 ± 4.6
Elongation at Break (ε_b_)			
Static	0.0959 ± 0.0082	0.1045 ± 0.0093	0.1182 ± 0.0108
5%	0.1518 ± 0.0125	0.1205 ± 0.0094	0.1094 ± 0.0077
10%	0.1173 ± 0.012	0.0954 ± 0.0089	0.0974 ± 0.0077

**Figure 6:**
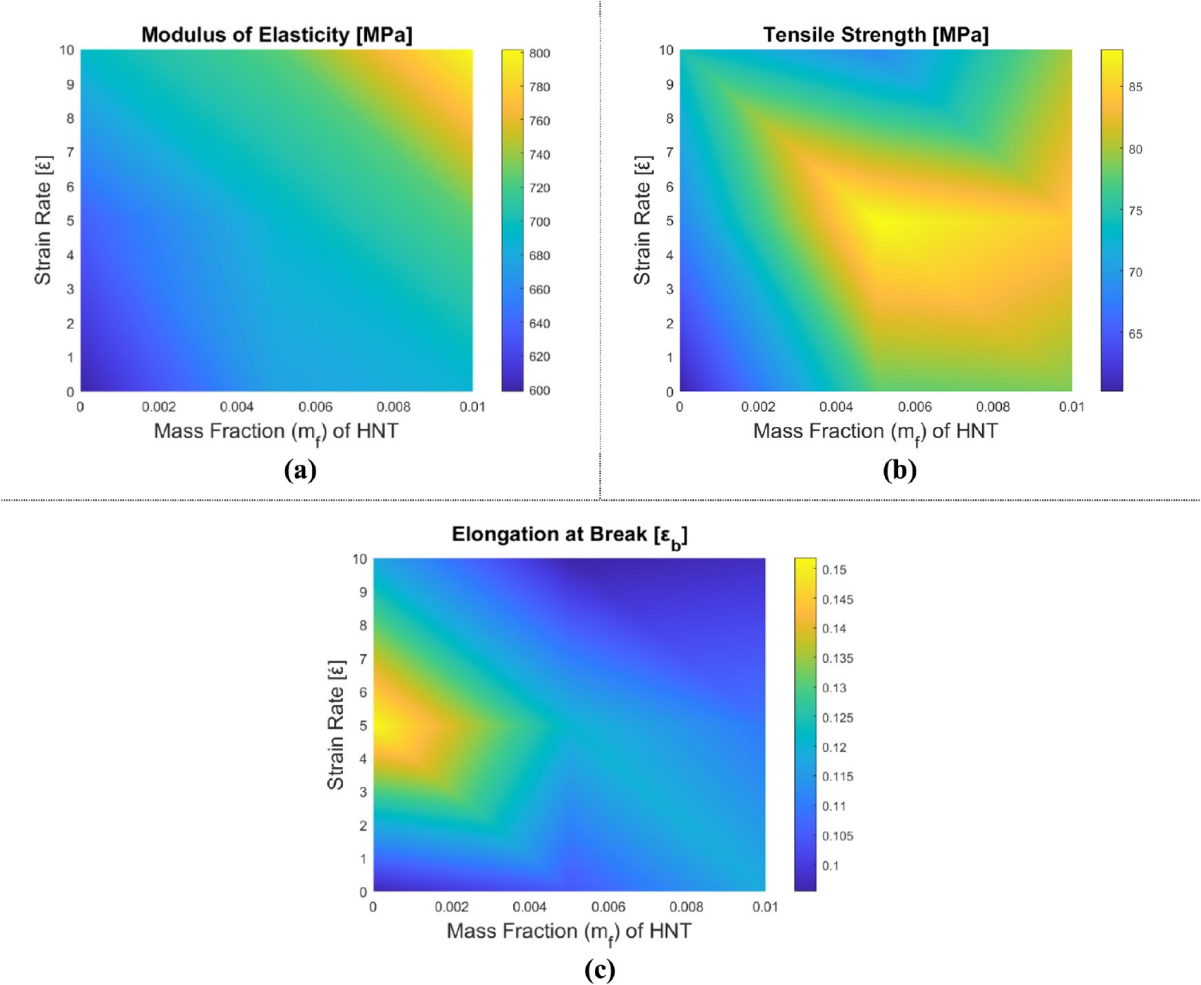
Variation of the mechanical properties of HNT-reinforced epoxy composite samples depending on the tensile test: (a) Elasticity modulus, (b) tensile strength, and (c) elongation at break.

**Figure 7:**
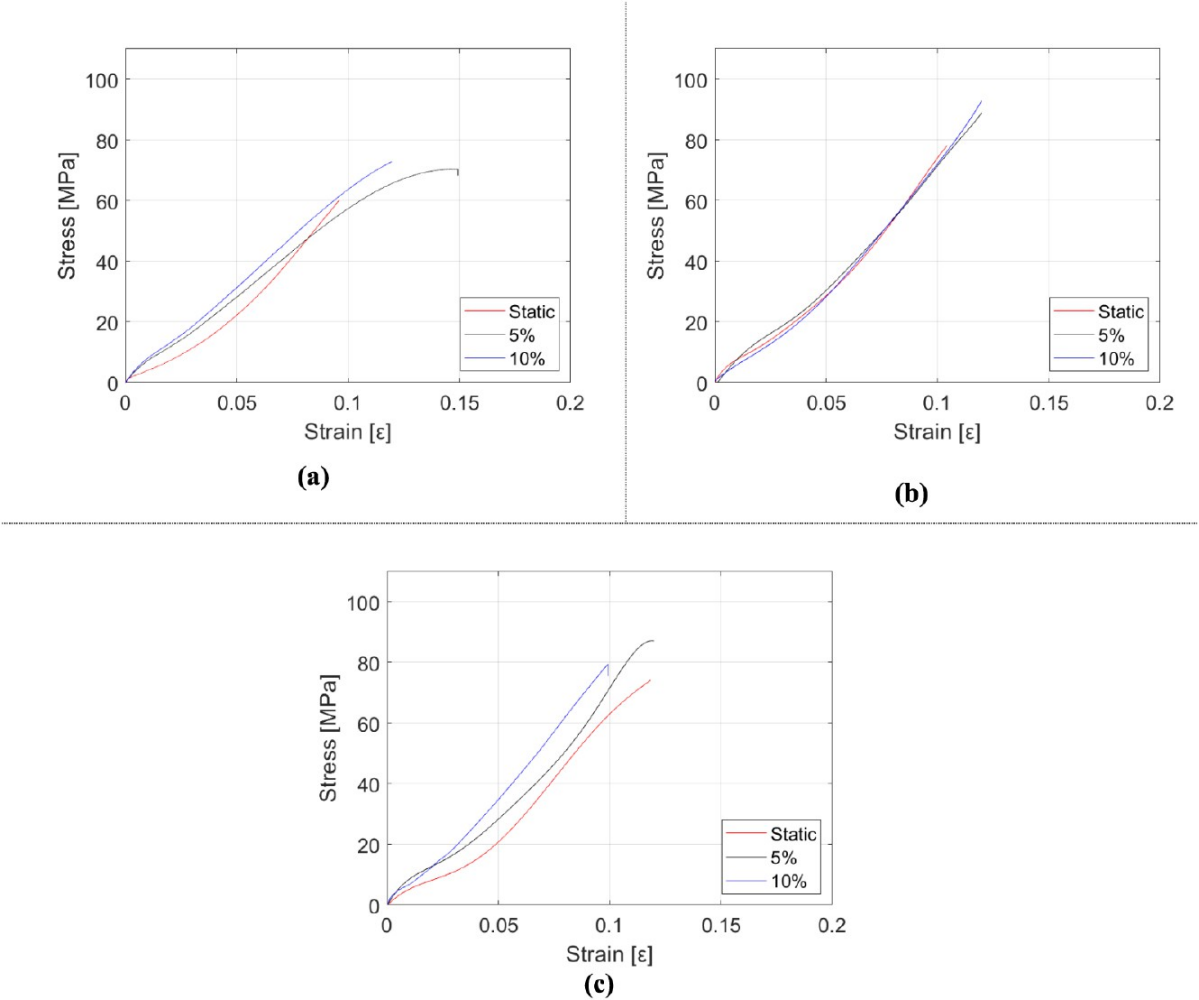
Stress–strain diagrams of HNT-reinforced epoxy composite samples depending on the strain rate. (a) epoxy, (b) m_f_ = 0.5% HNT-reinforced epoxy, and (c) m_f_ = 1% HNT-reinforced epoxy.

The tensile test results for CTBN rubber-reinforced epoxy samples are displayed in Table 4, Figures 8 and 9. Stress–strain plots are plotted with respect to the strain rate for each reinforcement combination in Figure 9. In contrast to HNT reinforcement, the elasticity modulus values of the material diminish as the mass fraction of the rubber increases. However, the influence of the strain rate on stiffness exhibits a pattern similar to that of the previous samples, with an observed rise in elasticity modulus values corresponding to increasing strain rates. The elongation at break values is higher with rubber inclusions compared to the pure epoxy control group. Furthermore, a comparison between the samples containing 5% and 10% rubber by weight within the epoxy matrix and the control group reveals a decrease in tensile strength values. Consequently, the rubber-reinforced epoxy composite demonstrates a weaker and more ductile behavior as rubber inclusions are introduced. The data highlight the trade-offs between stiffness, tensile strength, and ductility as CTBN rubber is incorporated into the epoxy matrix, showing the complicated effects of these factors in rubber-reinforced epoxy composites.

**Table 4:** Experimentally Characterized Mechanical Properties of Epoxy Reinforced with CTBN Rubber in the Tensile Test

RC	EP	R5	R10
Elasticity Modulus (E) [MPa]			
Static	598.9 ± 30.7	413.1 ± 31.6	370.1 ± 26.8
5%	635.6 ± 17.5	499.6 ± 39.1	450.2 ± 19.8
10%	693.7 ± 46.2	513.1 ± 16.0	467.9 ± 24.5
Tensile Strength (σ_t_) [MPa]			
Static	60.2 ± 6.7	84.8 ± 5.4	64.2 ± 5.8
5%	68.1 ± 4.6	80.4 ± 9.0	60.2 ± 10.9
10%	74.2 ± 5.6	73.7 ± 7.7	64.0 ± 4.6
Elongation at Break (ε_b_)			
Static	0.0959 ± 0.0082	0.1722 ± 0.0122	0.1416 ± 0.0094
5%	0.1518 ± 0.0125	0.1551 ± 0.0146	0.1195 ± 0.0092
10%	0.1173 ± 0.0120	0.1314 ± 0.0079	0.1507 ± 0.0130

**Figure 8:**
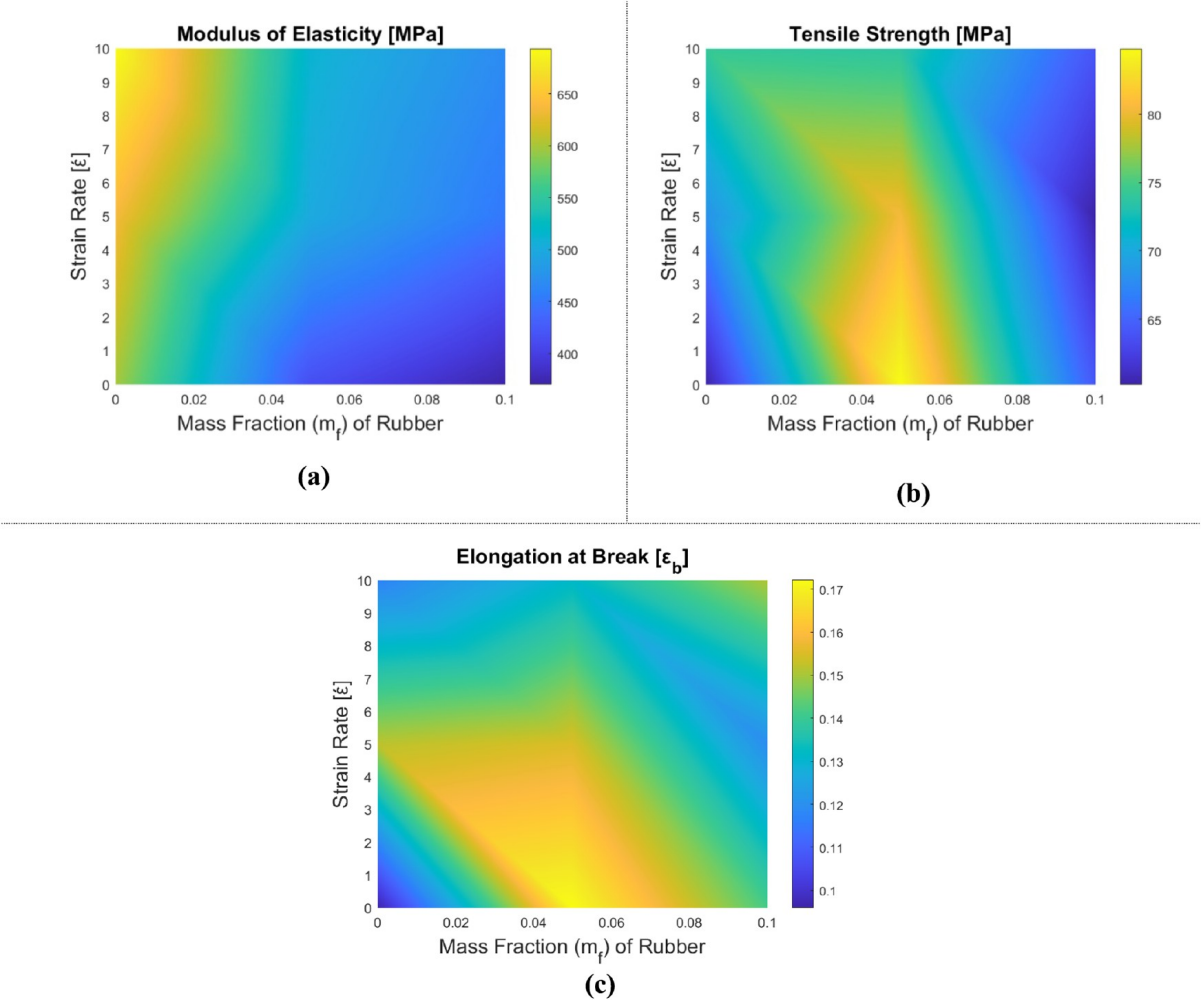
Variation of mechanical properties of rubber-reinforced epoxy composite samples depending on the tensile test: (a) elasticity modulus, (b) tensile strength, and (c) elongation at break.

**Figure 9:**
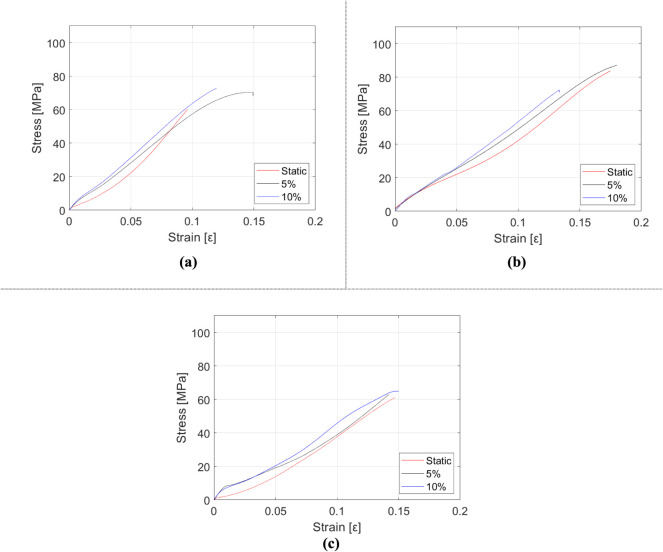
Stress–strain diagrams of rubber-reinforced epoxy composite samples depending on the strain rate: (a) epoxy, (b) m_f_ = 5% rubber-reinforced epoxy, and (c) m_f_ = 10% rubber-reinforced epoxy.

The synergistic effect of incorporating particles with distinct characteristics into the composite material is also investigated. The data obtained from the tests are presented in Figures 10, 11, and Table 5. In Figure 10, the mechanical property change of composites with respect to the total reinforcement mass fraction is evaluated. Figure 11 displays stress–strain plots of each manufactured sample for three strain rates. In Table 5, in order to compare values and evaluate the synergistic effect, the elasticity modulus, tensile strength, and elongation at break values are given. While HNT reinforcement contributes to rigidity, rubber inclusions provide ductility. Interpreting the results can be more complex when both rigidity and ductility reinforcements are employed concurrently. First, it is evident that the material’s stiffness increases with the rising strain rate. The stiffness of rubber- and HNT-reinforced composites is observed to decrease in comparison to the pure epoxy control group during tensile testing, which can be attributed to the rubber’s influence. Furthermore, an enhancement in elongation at break values is noted, and the material exhibits ductile behavior. The tensile strength is found to demonstrate varying responses based on strain rate and reinforcement type. This analysis highlights the complicated characteristics of stiffness, ductility, and tensile strength in composites featuring both HNT and rubber reinforcements, providing a more comprehensive perspective into the potential benefits and challenges of combining these materials.

**Table 5:** Experimentally Characterized Mechanical Properties of Epoxy Reinforced with HNT and CTBN Rubber in the Tensile Test

RC	PE	H05R5	H05R10	H1R5	H1R10
Elasticity Modulus (E) [MPa]					
Static	598.9 ± 30.7	468.8 ± 19.6	430.8 ± 33.3	502.7 ± 31.0	458.8 ± 24.3
5%	635.6 ± 17.5	485.3 ± 16.3	518.0 ± 25.9	556.5 ± 26.7	521.1 ± 18.7
10%	693.7 ± 46.2	592.2 ± 27.6	537.9 ± 36.9	682.3 ± 42.5	564.6 ± 29.4
Tensile Strength (σ_t_) [MPa]					
Static	60.2 ± 6.7	65.7 ± 7.2	65.8 ± 6.2	79.9 ± 7.8	79.1 ± 4.7
5%	68.1 ± 4.6	65.9 ± 5.7	69.8 ± 9.9	76.8 ± 8.5	65.8 ± 8.2
10%	74.2 ± 5.6	65.0 ± 6.1	67.5 ± 8.5	71.2 ± 6.8	62.2 ± 9.8
Elongation at Break (ε_b_)					
Static	0.0959 ± 0.0082	0.128 ± 0.0137	0.1242 ± 0.0134	0.1379 ± 0.0107	0.1567 ± 0.0138
5%	0.1518 ± 0.0125	0.1113 ± 0.0143	0.1328 ± 0.0076	0.1351 ± 0.0089	0.1190 ± 0.0084
10%	0.1173 ± 0.0120	0.1275 ± 0.0083	0.1326 ± 0.0116	0.1166 ± 0.0114	0.1028 ± 0.0093

**Figure 10:**
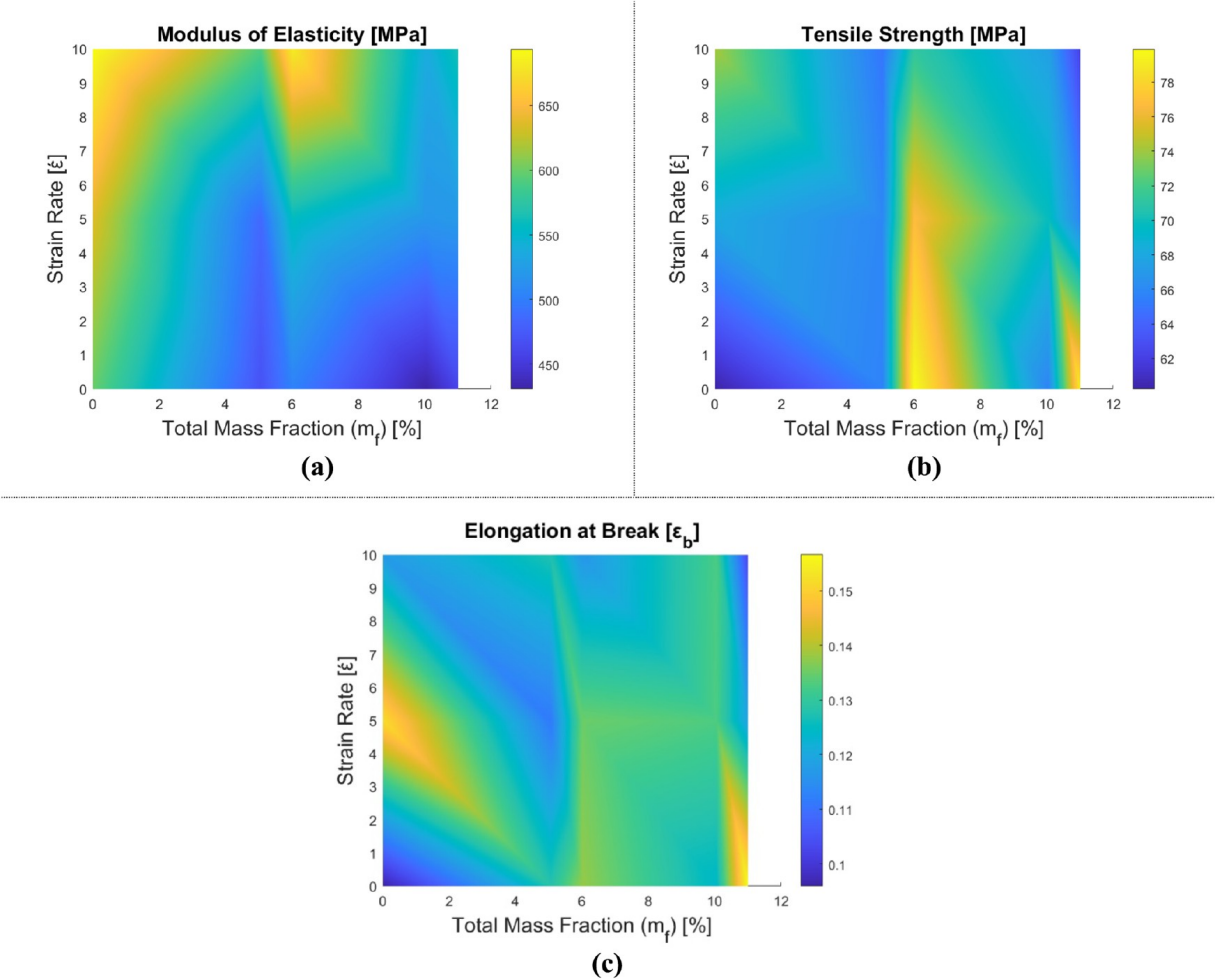
Variation of mechanical properties of HNT and rubber-reinforced epoxy composite samples depending on the tensile test: (a) elasticity modulus, (b) tensile strength, and (c) elongation at break.

**Figure 11:**
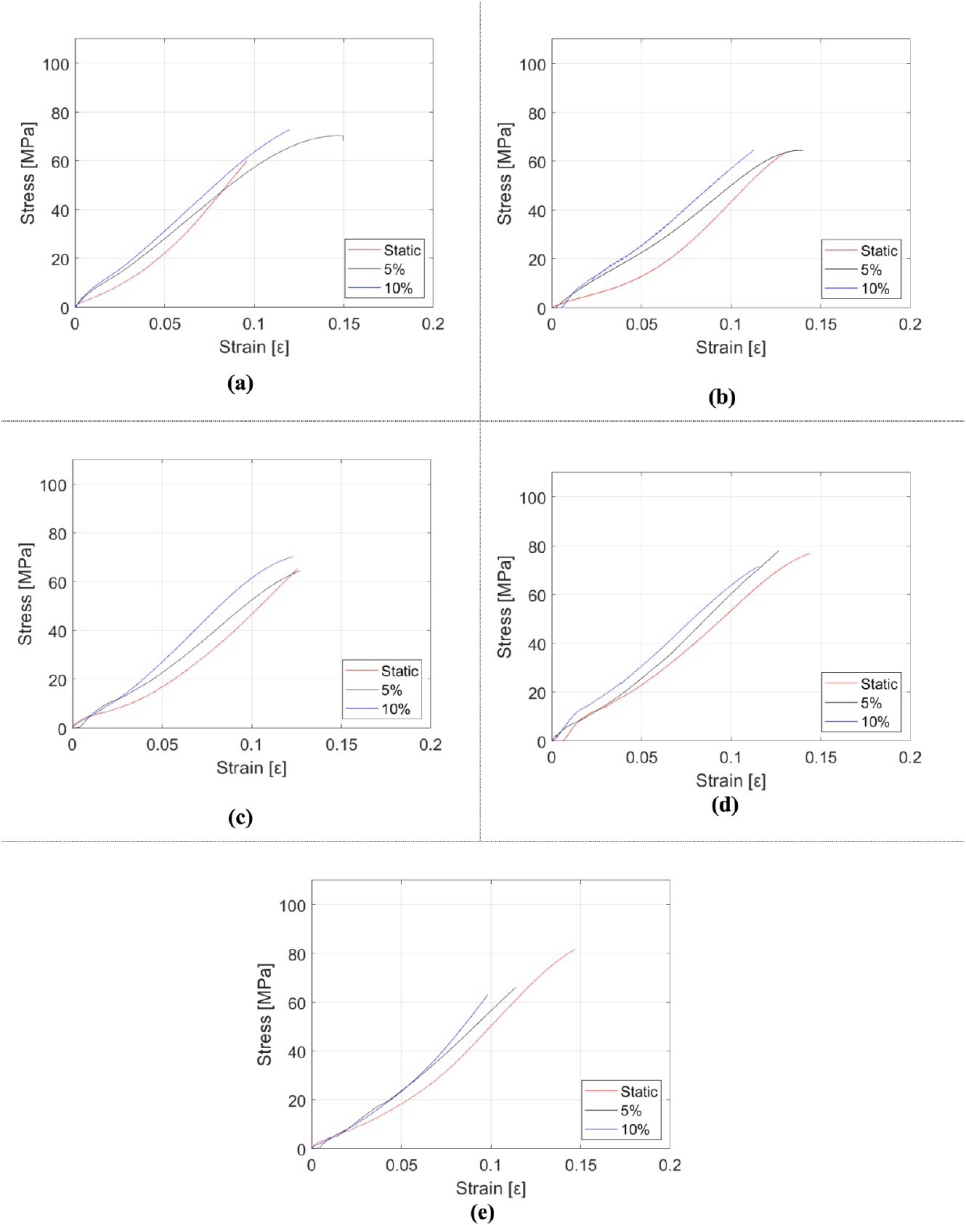
Stress–strain diagrams of HNT and rubber-reinforced epoxy composite samples depending on the strain rate: (a) epoxy, (b) m_f_ = 5% rubber and m_f_ = 0.5% HNT-reinforced epoxy, (c) m_f_ = 10% rubber and m_f_ = 0.5% HNT-reinforced epoxy, (d) m_f_ = 5% rubber and m_f_ = 1% HNT-reinforced epoxy, and (e) m_f_ = 10% rubber and m_f_ = 1% HNT-reinforced epoxy.

#### 4.1.2 Bending Behavior of Nanocomposites

This section presents the results of the three-point bending tests, which aim to determine the strain rate-dependent mechanical characterization of the epoxy matrix composites. Elasticity modulus (E), flexural strength (σ_ult_), and elongation at break (ε_b_) values obtained from three-point bending tests at different strain rates and with different reinforcement types are given.

Figure 12 displays the variations in the mechanical properties of HNT-reinforced epoxy matrix nanocomposite samples, dependent on the strain rate and HNT mass fraction. In Table 6, the obtained results are given in numbers, and in Figure 13, the stress–strain relationship of HNT-reinforced composites under bending is marked. The stiffness of the composites is observed to increase with HNT reinforcement, and a similar trend emerges with the rising strain rate. Conversely, elongation values at break diminish with escalating strain rates, reaching a minimum at the highest strain rate and m_f_ = 0.5% HNT. Elongation at break experiences an increase at m_f_ = 1% HNT compared to m_f_ = 0.5% HNT. This can be elucidated by examining the stress state in the material. HNT reinforcements induce triaxial stress concentrations within the epoxy matrix but also enhance the material’s load-bearing capacity. As a result, in the case of m_f_ = 0.5% HNT reinforcement, a partially brittle behavior is observed due to the formation of stress concentrations. However, as the mass fraction of HNT increases, its contribution to load-carrying ability also rises, delaying crack formation resulting from stress concentrations. Consequently, the subsequent delay in damage is an expected outcome. Thus, further investigations on the weight percentage of HNT in the epoxy matrix can be assessed to achieve a more ductile behavior concerning material performance and properties.

**Table 6:** Experimentally Characterized Mechanical Properties of Epoxy Reinforced with HNT in the Three-Point Bending Test

RC	PE	H05	H1
Elasticity Modulus (E) [MPa]			
Static	2410.8 ± 71.6	2648.6 ± 54.3	2827.0 ± 102.4
5%	2627.7 ± 90.7	2765.1 ± 82.1	2998.8 ± 75.4
10%	2807.0 ± 115.0	2922.1 ± 140.6	3121.3 ± 65.8
Flexural Strength (σ_ult_) [MPa]			
Static	101.1 ± 7.3	81.9 ± 11.9	100.8 ± 12.4
5%	103.3 ± 10.6	88.8 ± 8.8	84.7 ± 13.2
10%	105.1 ± 9.8	74.0 ± 9.6	91.3 ± 6.8
Elongation at Break (ε_b_)			
Static	0.0487 ± 0.00219	0.03299 ± 0.00230	0.0342 ± 0.00218
5%	0.0436 ± 0.00265	0.0356 ± 0.00304	0.0311 ± 0.00296
10%	0.0393 ± 0.00235	0.0264 ± 0.00271	0.0337 ± 0.00213

**Figure 12:**
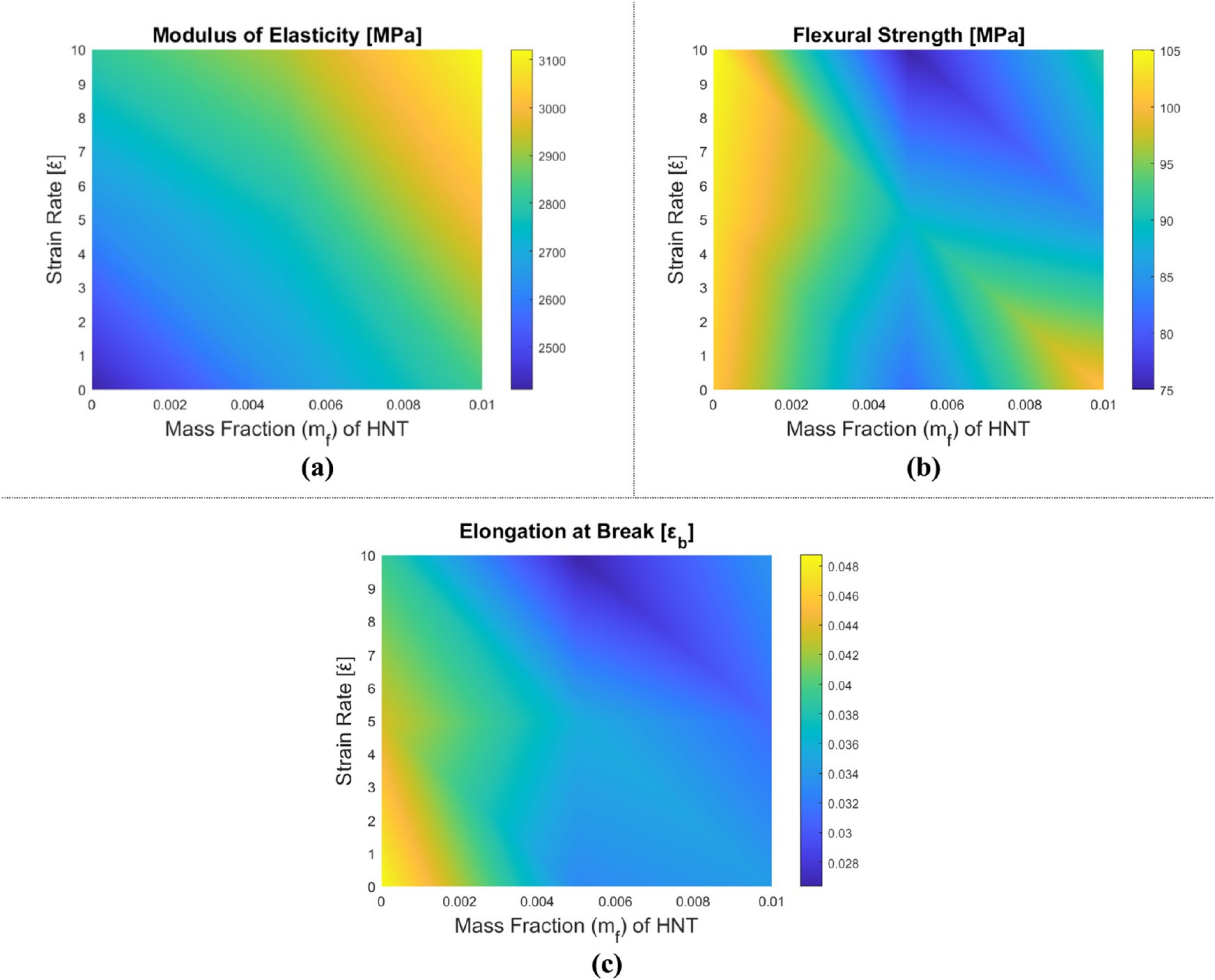
Variation of mechanical properties of HNT-reinforced epoxy composite samples in the three-point bending test: (a) elasticity modulus, (b) flexural strength, and (c) elongation at break.

**Figure 13:**
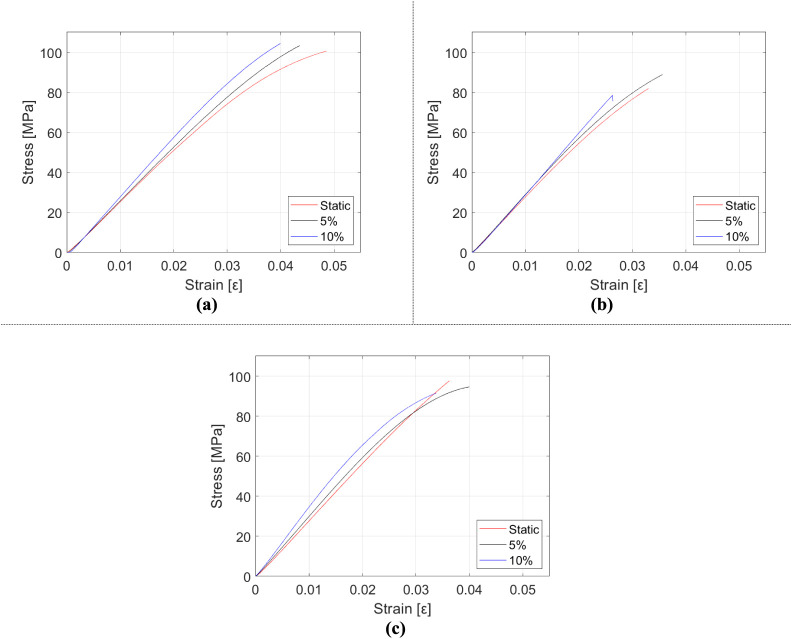
Stress–strain diagrams of HNT-reinforced epoxy composite samples depending on the strain rate: (a) epoxy, (b) m_f_ = 0.5% HNT-reinforced epoxy, and (c) m_f_ = 1% HNT-reinforced epoxy.

The three-point bending test results for CTBN rubber-reinforced epoxy samples are presented in Table 7, Figures 14 and 15. Table 7 represents mechanical properties in numbers in order to investigate the effect of rubber on bending properties. Figure 14 represents mechanical behavior change with respect to the reinforcement ratio, and Figure 15 indicates the stress–strain behavior of manufactured composites regarding strain rate. The mechanical properties of rubber-doped samples exhibit notable differences compared to HNT-doped samples. The most significant contrast lies in the fact that increasing rubber content leads to a reduction in both flexural strength and rigidity. Elongation at break values rise with rubber inclusions, and samples containing rubber inclusions demonstrate higher elongation at break values than their HNT-reinforced counterparts, as well as unreinforced epoxy. Furthermore, stiffness is observed to increase in tandem with strain rate escalation. Although the material weakens with a greater concentration of rubber particles, the material’s ductility experiences a considerable enhancement.

**Table 7:** Experimentally Characterized Mechanical Properties of Epoxy Reinforced with CTBN Rubber in the Three-Point Bending Test

RC	PE	R5	R10
Elasticity Modulus (E) [MPa]			
Static	2410.8 ± 71.6	2383.8 ± 124.6	2083.5 ± 61.5
5%	2627.7 ± 90.7	2474.7 ± 82.7	2350.3 ± 73.9
10%	2807.0 ± 115.0	2587.2 ± 58.6	2413.1 ± 55.8
Flexural Strength (σ_ult_) [MPa]			
Static	101.1 ± 7.3	92.7 ± 12.1	81.1 ± 7.5
5%	103.3 ± 10.6	98.5 ± 10.3	72.2 ± 6.2
10%	105.1 ± 9.8	89.9 ± 11.1	87.8 ± 11.0
Elongation at Break (ε_b_)			
Static	0.0487 ± 0.00219	0.0479 ± 0.00337	0.0384 ± 0.00220
5%	0.0436 ± 0.00265	0.0514 ± 0.00291	0.0382 ± 0.00301
10%	0.0393 ± 0.00235	0.0403 ± 0.00289	0.0448 ± 0.00346

**Figure 14:**
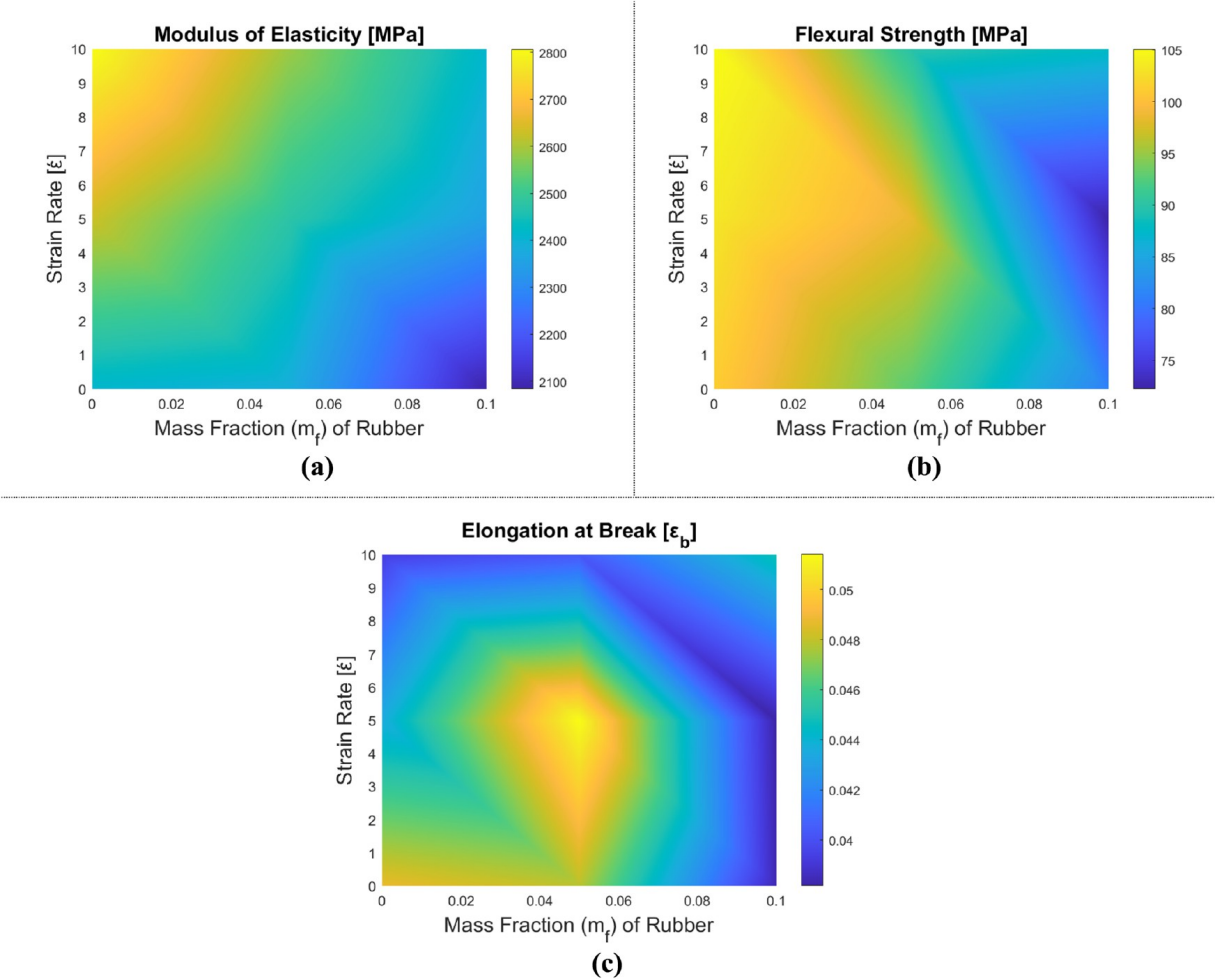
Variation of mechanical properties of rubber-reinforced epoxy composite samples: (a) elasticity modulus, (b) flexural strength, and (c) elongation at break.

**Figure 15:**
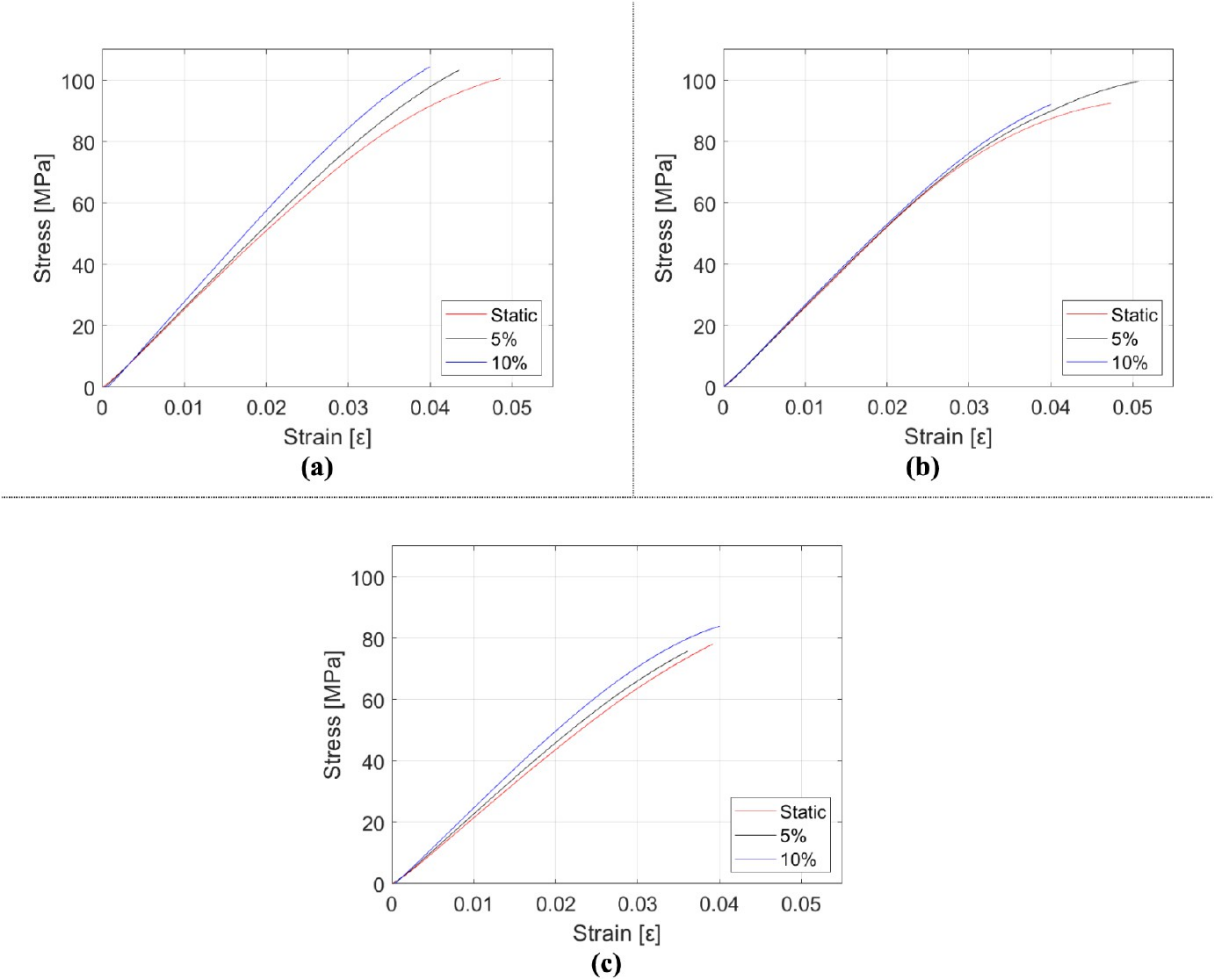
Stress–strain diagrams of rubber-reinforced epoxy composite samples depending on the strain rate: (a) epoxy, (b) m_f_ = 5% rubber-reinforced epoxy, and (c) m_f_ = 10% rubber-reinforced epoxy.

The mechanical properties of epoxy matrix composites reinforced with both HNT and CTBN rubber are also investigated to explore their synergistic effect. The data obtained from this analysis can be found in Figures 16,17, and Table 8. While Figure 16 represents the change in mechanical behavior with respect to the total reinforcement ratio, Table 8 represents this change numerically. In Figure 17, stress–strain behavior and the synergetic and strain rate effects are plotted. The synergy between HNT and CTBN rubber results in a more complex alteration of the material behavior. Consequently, stiffness and flexural strength are seen to increase with rising strain rates, while a decline in elongation at break values is observed. Furthermore, in the epoxy composite with m_f_ = 10% rubber and m_f_ = 0.5% HNT, a decrease in stiffness is noticed compared to pure epoxy. Rigidity increases with a higher HNT content and lower rubber ratio in the other samples. Examining the flexural strength values, it is evident that all samples display values lower than those of pure epoxy, as anticipated. The elongation values at break of these samples are higher than those observed in HNT-doped epoxy nanocomposites.

**Table 8:** Experimentally Characterized Mechanical Properties of Epoxy Reinforced with HNT and CTBN Rubber in the Three-Point Bending Test

RC	PE	H05R5	H05R10	H1R5	H1R10
Elasticity Modulus (E) [MPa]					
Static	2410.8 ± 71.6	2312.0 ± 112.3	2293.9 ± 76.4	2538.4 ± 53.1	2442.7 ± 72.9
5%	2627.7 ± 90.7	2552.7 ± 84.8	2436.6 ± 56.3	2675.5 ± 68.6	2568.2 ± 94.8
10%	2807.0 ± 115.0	2627.6 ± 60.8	2508.1 ± 53.9	2925.3 ± 63.8	2713.1 ± 67.3
Flexural Strength (σ_ult_) [MPa]					
Static	101.1 ± 7.3	87.3 ± 9.4	86.7 ± 11.2	86.8 ± 6.3	84.1 ± 6.8
5%	103.3 ± 10.6	88.7 ± 6.3	89.8 ± 9.2	93.0 ± 7.3	87.6 ± 2.8
10%	105.1 ± 9.8	83.6 ± 7.9	98.4 ± 7.8	90.5 ± 5.9	84.6 ± 7.0
Elongation at Break (ε_b_)					
Static	0.0487 ± 0.00219	0.0522 ± 0.00220	0.0476 ± 0.00261	0.0453 ± 0.00213	0.0428 ± 0.00224
5%	0.0436 ± 0.00265	0.0431 ± 0.00300	0.0415 ± 0.00322	0.0485 ± 0.00151	0.0441 ± 0.00201
10%	0.0393 ± 0.00235	0.0366 ± 0.00340	0.0493 ± 0.00251	0.0378 ± 0.00314	0.0407 ± 0.00218

**Figure 16:**
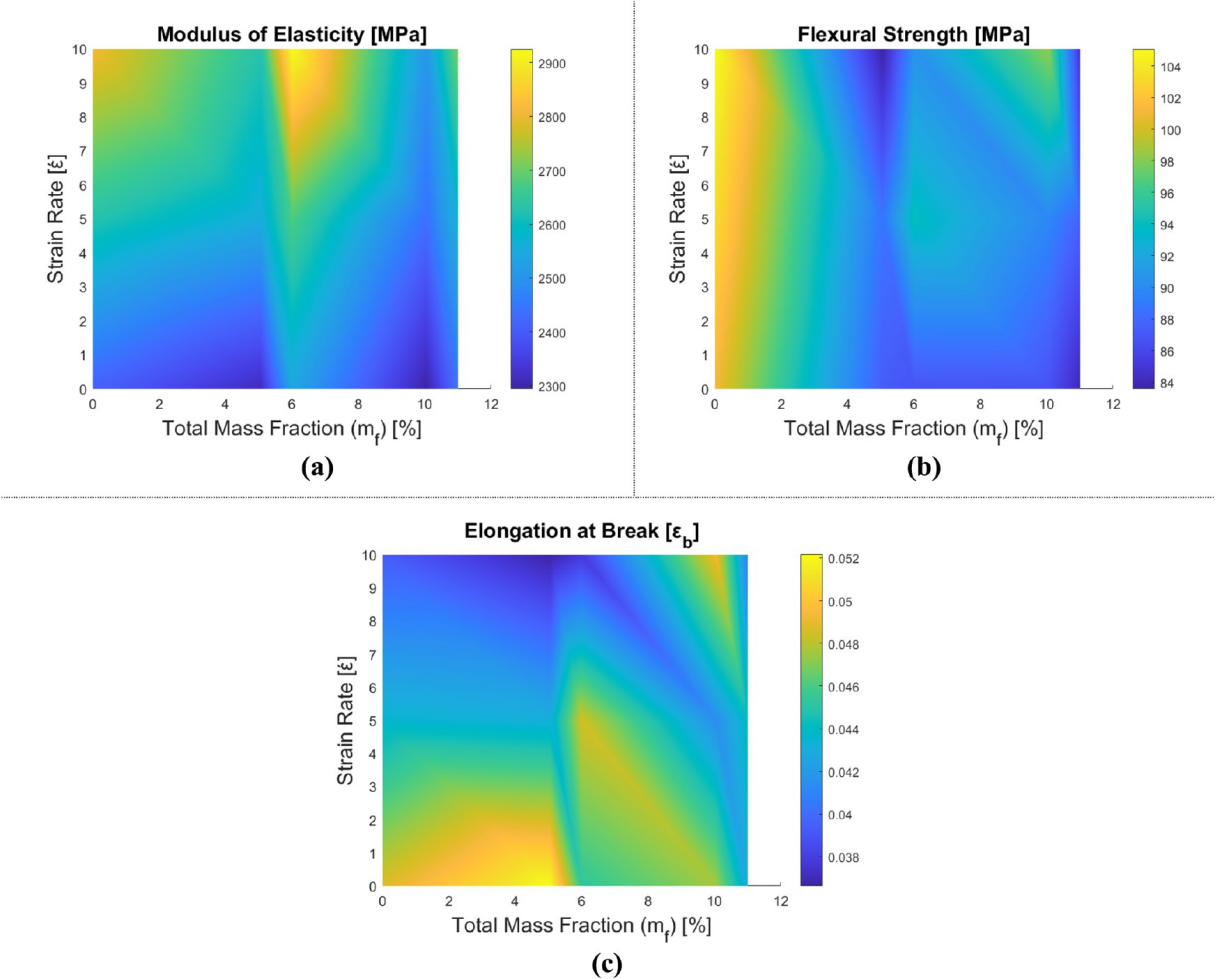
Variation of mechanical properties of rubber and HNT-reinforced epoxy composite samples: (a) elasticity modulus, (b) flexural strength, and (c) elongation at break.

**Figure 17:**
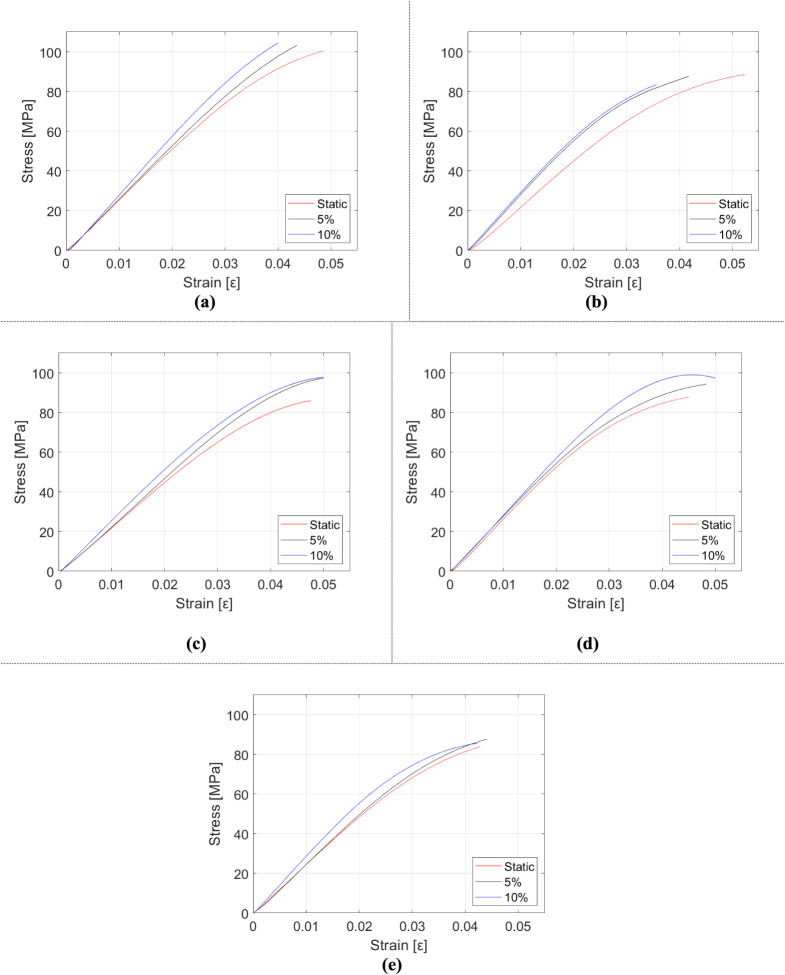
Stress–strain diagrams of HNT and rubber-reinforced epoxy composite samples depending on the strain rate: (a) epoxy, (b) m_f_ = 5% rubber and m_f_ = 0.5% HNT-reinforced epoxy, (c) m_f_ = 10% rubber and m_f_ = 0.5% HNT-reinforced epoxy, (d) m_f_ = 5% rubber and m_f_ = 1% HNT-reinforced epoxy, and (e) m_f_ = 10% rubber and m_f_ = 1% HNT-reinforced epoxy.

#### 4.1.3 Discussion of the Tensile and Three-Point Bending Behavior of Nanocomposites

Evaluating the experimental results focusing on the three-point bending tests and tensile tests, it is clearly seen that the mechanical properties of the tested materials are significantly different. This is a common phenomenon, as the measured material properties display significant differences depending on the test method. There are noticeable differences even between four-point bending and three-point bending tests,.^76,77^

The significant differences observed in the modulus of elasticity, elongation at break, and strength values for the same materials (epoxy- and epoxy-based nanocomposites) in flexural and tensile tests can be attributed to the distinct nature of the applied loads and the materials’ response to these loads. There are studies in the literature presenting test results with similar differences that belong to epoxy,.^78,79^

In tensile tests, the material is subjected to unidirectional positive stress, leading to a uniaxial tensile loading. This test evaluates the behavior of the material under tensile forces, which tend to stretch and elongate the material. Conversely, in flexural tests, such as the three-point bending test, the material experiences both tensile and compressive loads simultaneously. The test specimen undergoes simple bending, resulting in a complex coexistence of behavior under both compression and tension.

The material’s behavior under tensile loads may differ significantly from that under compressive loads, resulting in different outcomes between the tensile and flexural tests.^80^ For instance, some materials might be more resistant to tension and more prone to elongation, whereas others might be more resistant to bending or compression,.^77,81^ Additionally, the stress distribution within the material varies between these two tests, potentially leading to different failure mechanisms and mechanical responses,.^27,82,83^

Moreover, the epoxy matrix’s inherent brittleness and its response to the addition of HNT and rubber inclusions contribute to the discrepancies observed in the test results. The HNT additive increases the stiffness of the composite, while rubber inclusions enhance its ductility,.^17,19,27,84^ As a result, the combined effect of these reinforcements creates a complex interaction between stiffness and ductility in the material, which may yield differently under tensile and flexural loads.^79,85^

As anticipated, the outcomes of the three-point bending tests and tensile tests generally exhibit some parallelism, and they remain consistent within themselves, evaluating with reinforcement dependence. Nevertheless, the elasticity modulus and tensile strength values obtained from the tensile tests show considerable differences. The primary reason for this significant discrepancy is the distinct mechanical behavior of the epoxy matrix under tensile and compressive loads.

While the tensile test evaluates the material’s response to unidirectional positive stress by applying a uniaxial tensile load, the three-point bending test exposes the test specimen to both tensile and compressive loads simultaneously through simple bending. This results in a complex nature and behavior under both compression and tension. Consequently, the experimental material behavior deviates from the idealized expectations, even though it is assumed that the two experiments would yield similar values in theory.

Therefore, the observed difference between the tensile and three-point bending test results is a common issue that arises from the complex nature of material behavior under different loading conditions.

#### 4.1.4 Fracture Surfaces of Damaged Samples

For a detailed inspection of the fracture surfaces of the damaged samples, the Nikon SMZ800 stereo microscope is used. It comes with a camera, which helps capture high-resolution images for thorough analysis. First, samples subjected to three-point bending tests are evaluated within the scope of this section. The fracture surfaces of HNT-reinforced, CTBN rubber-reinforced, and both HNT and CTBN rubber-reinforced damaged samples can be seen in Figure 18. Figure 18 (a) illustrates the fracture surface images of tensile testing on HNT-reinforced epoxy samples. Figure 18c displays the fracture surface images of tensile testing on epoxy samples reinforced with rubber. Finally, Figure 18b represents the fracture surface images of tensile testing on epoxy samples reinforced with both HNT and rubber. Epoxy samples reinforced with HNT and rubber are chosen because HNT increases stiffness and reduces ductility, while rubber has the opposite effect. Fracture surfaces of the materials become clearer and sharper as the level of HNT reinforcement increases and the rubber reinforcement ratio decreases. This can be attributed to the brittleness effect of the HNT reinforcement.

**Figure 18:**
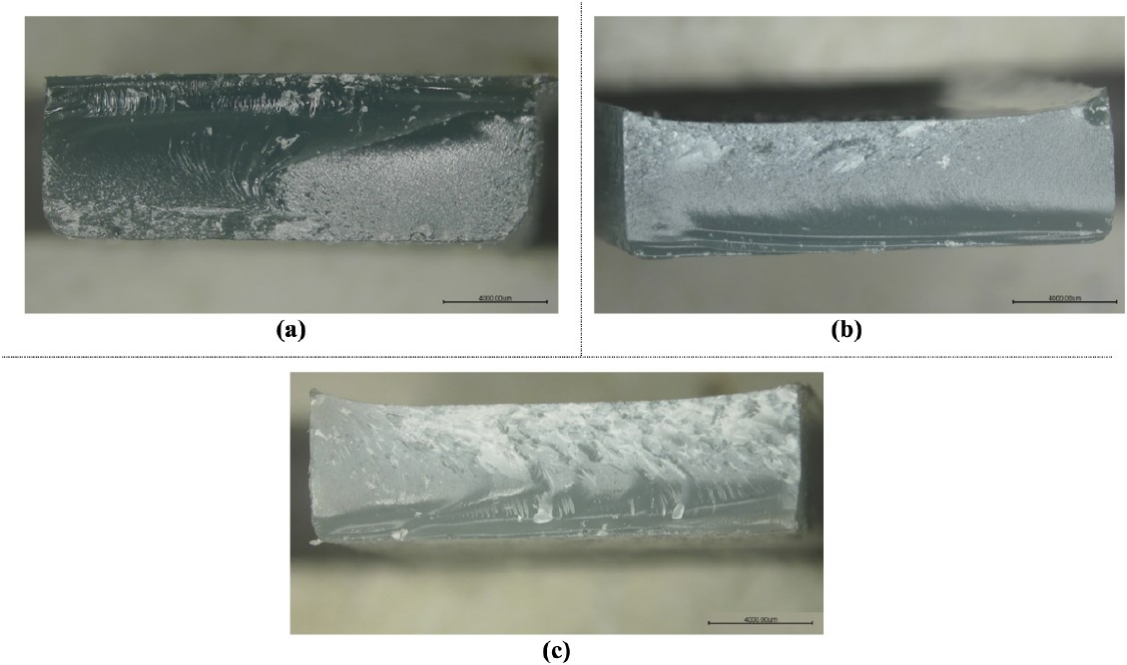
Fracture surface images of the damaged three-point bending samples: (a) HNT-reinforced, (b) HNT and rubber-reinforced, and (c) rubber-reinforced.

Figure 19 represents fracture surface images of the tensile test specimens. Similar to Figure 18, in Figure 19, the effect of the nanoparticle reinforcement type on the ductility of the sample is examined from the fractured surface images. Fracture surface image results in Figure 19 tend to be similar to those from three-point bending test samples. In Figure 19a, the fracture surfaces of the epoxy samples reinforced with halloysite nanotubes (HNT) are illustrated, highlighting the characteristic failure patterns due to the presence of HNT. Figure 19b shows the fracture surfaces of epoxy samples reinforced with both HNT and rubber, demonstrating the combined effects of these reinforcements on the fracture behavior. Finally, Figure 19c displays the fracture surfaces of epoxy samples reinforced with rubber, showing the effect of the rubber reinforcement. These images provide valuable information about the influence of different reinforcement materials on the mechanical properties and fracture mechanisms of epoxy composites. Samples containing HNT nanoparticles, rubber reinforcement, and those reinforced with both HNT and rubber are selected for analysis. HNT enhances stiffness but diminishes ductility, whereas rubber exhibits the opposite behavior. As the proportion of HNT reinforcement rises and that of rubber decreases, the fracture surfaces of the materials become clearer and sharper. This phenomenon is likely due to the increased brittleness caused by the HNT reinforcement.

**Figure 19:**
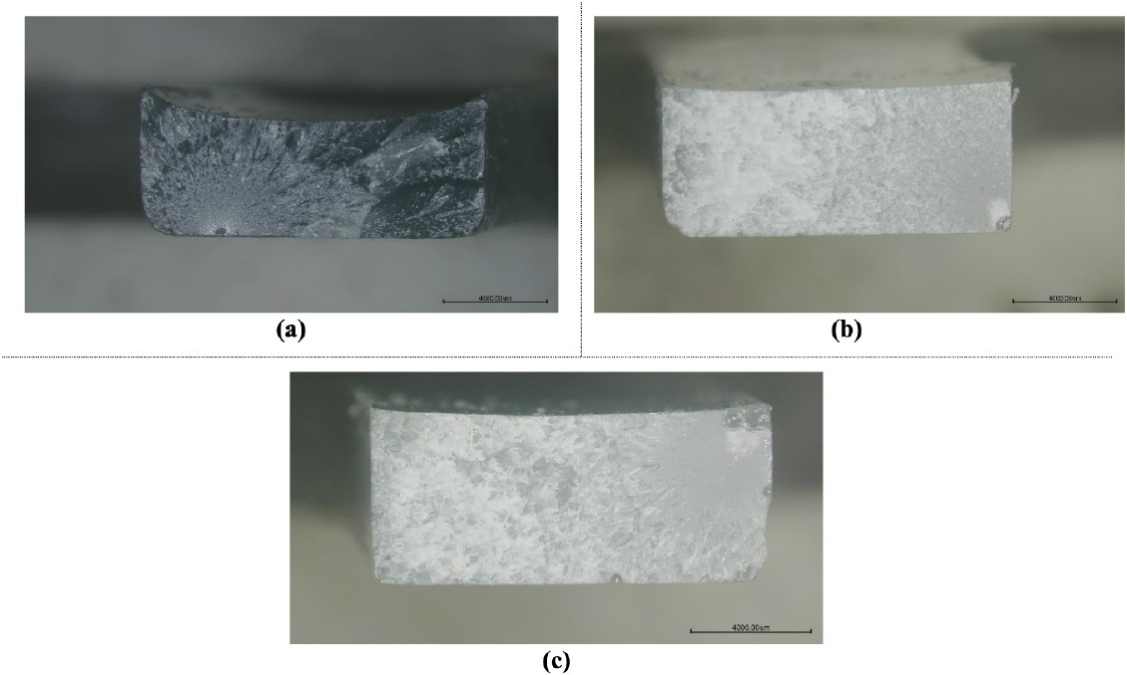
Fracture surface images of the damaged tensile test samples: (a) HNT-reinforced, (b) HNT and rubber-reinforced, and (c) rubber-reinforced.

#### 4.1.5 Fracture Energy of Nanocomposites

After completing the mechanical characterization based on the strain rate, the fracture behavior of these materials is also explored, and the results are presented. In this regard, Charpy impact tests are conducted six times for each composite material combination, and a column chart is created by averaging the experimental outcomes. The resulting graph is displayed in Figure 20.

**Figure 20:**
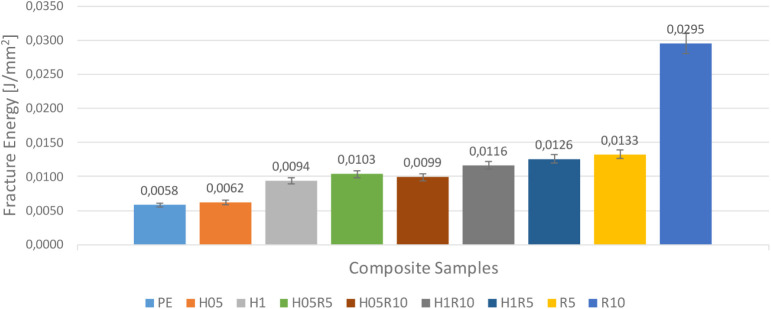
Fracture energy of the manufactured composite samples.

It is evident from the graph that both HNT and rubber inclusions increase the fracture energy of the composite, with the lowest fracture energy value being observed in pure epoxy samples. A modest increase in fracture energy is observed in the m_f_ = 0.5% HNT-reinforced epoxy nanocomposite, while the most significant increase occurs in the m_f_ = 10% rubber-reinforced epoxy. Based on these findings, it can be concluded that both rubber and HNT contribute to enhancing the fracture energy.

Conversely, using rubber and HNT particles together as reinforcing materials results in an intermediate behavior between HNT-only and rubber-only reinforced composites. Thus, it becomes apparent that the synergistic effect of these two particles, which individually exhibit similar effects on the material’s fracture energy, does not generate any unpredictable or complex outcomes.

In Figure 21, fracture surfaces of Charpy impact test samples from each material group, featuring different reinforcement types with contrasting characteristics in terms of ductility and stiffness, are presented. Specifically, Figure 21a displays HNT-reinforced epoxy samples, while Figure 21c showcases epoxy samples augmented with rubber, and Figure 21b represents both HNT- and rubber-reinforced ones. The selection criteria for these samples are based on the observed effects: HNT enhances stiffness and diminishes ductility, whereas rubber exhibits the opposite trend. The images reveal distinct differences between the fracture surfaces of these materials. The fracture surface of the HNT-reinforced epoxy appears brighter and more reflective, with less deformation or warping in the cross-section. This characteristic can be attributed to the heightened stiffness imparted by the HNT particles, which effectively enhance the composite’s overall stiffness.

**Figure 21:**
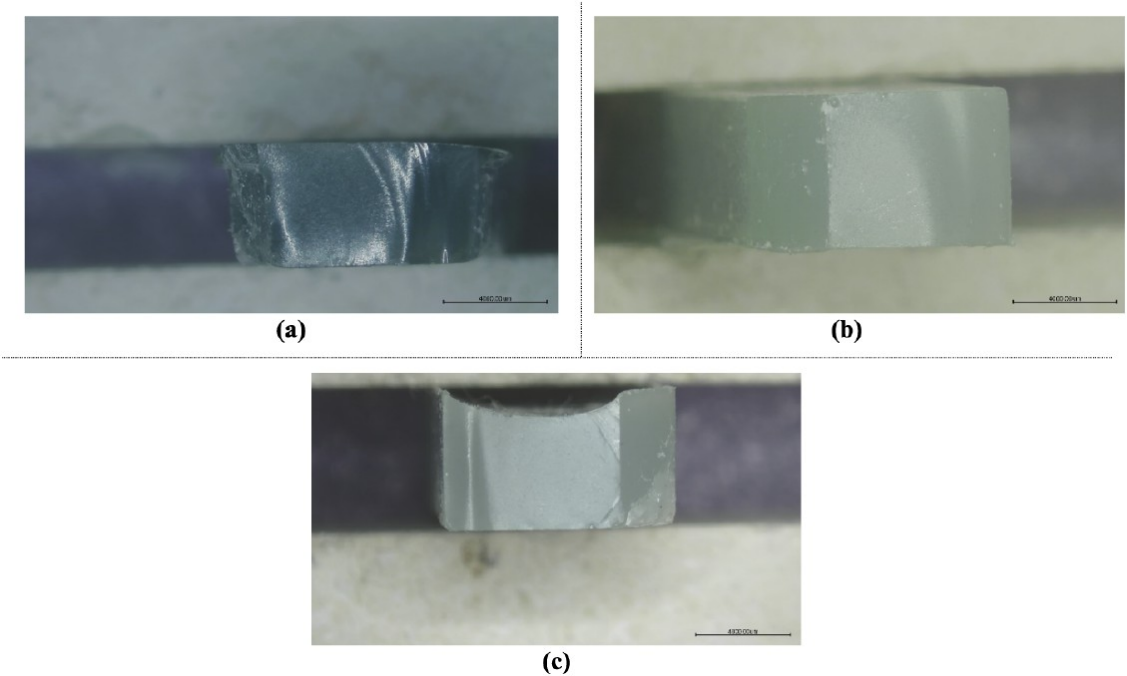
Fracture surface images of the damaged Charpy impact test samples: (a) HNT-reinforced, (b) HNT and rubber-reinforced, and (c) rubber-reinforced.

### 4.2 Scanning Electron Microscope Images

SEM images for each material type are given and evaluated in this section. Result images are divided into groups according to reinforcement types and are considered in sections.

#### 4.2.1 HNT-Reinforced Epoxy Samples

Figure 22 shows SEM images of unreinforced epoxy and 0.5% and 1% HNT-reinforced epoxy/HNT samples. Comparing Figures 22a and b, it is clearly seen that the surface of the epoxy sample is more homogeneous and smoother than the surfaces of the HNT-doped epoxy. At low mass fractions of HNT (0.5% HNT), HNT shows good dispersion in the epoxy matrix. In addition, compared to other HNT/epoxy samples, the presence of individual HNTs in the matrix is more clearly seen in the 0.5% HNT-reinforced epoxy sample. When the HNT content in the epoxy is 1%, the surface roughness of the material and the visibility of the HNT particles on the surface of the material increase.

**Figure 22:**
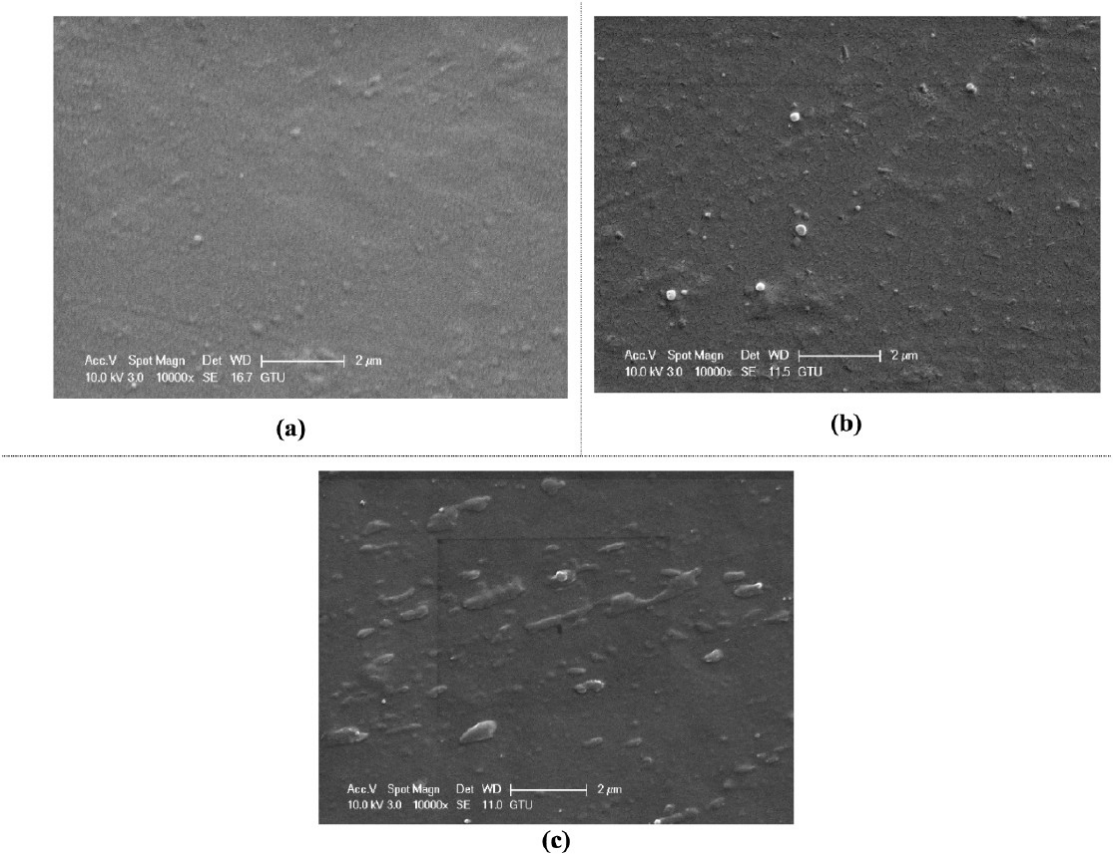
SEM images of epoxy/HNT samples reinforced with different amounts of HNT: (a) unreinforced epoxy, (b) 0.5% HNT, and (c) 1% HNT.

Similarly, there is a significant variation in the distribution of HNT depending on the mass fraction of HNT particles in the epoxy resin (from m_f_ = 2.5% to 6.5%).^86^ This can be related to the greater HNT presence, leading to a different nature of dispersion in the epoxy due to increased viscosity. The SEM images confirm that the employed manufacturing procedure successfully achieved a uniform dispersion of HNT particles at lower mass fractions (0.5%). At 0.5% HNT, individual nanotubes are well distributed throughout the matrix with minimal agglomeration. As expected from nanocomposite processing theory, increasing the reinforcement content to 1% HNT results in slightly increased heterogeneity due to elevated viscosity, which is consistent with findings in the literature.^87^ Nevertheless, no severe agglomeration or critical defects that would compromise the mechanical properties are observed.

#### 4.2.2 Rubber-Reinforced Epoxy Samples

Figure 23 shows SEM images of plain epoxy and 0.5% rubber-reinforced epoxy/rubber samples. With the inclusion of rubber in the epoxy, the material surfaces have become rougher. The presence of circular inclusions up to approximately 1 μm on the surface of the m_f_ = 5% rubber-reinforced sample is visible. On the other hand, when the amount of rubber inclusions in the epoxy increases, the material surface becomes a highly heterogeneous structure, the particles exhibit a more uneven distribution, and some particles larger than 1 μm are seen. The reason for this trend of heterogeneity and more uneven distribution can be related to the increased viscosity of the polymer, which directly affects the manufacturing of the composites, as explained earlier. For rubber-reinforced samples, the liquid nature of Albipox 1000 (which contains CTBN rubber) facilitates easier mixing and dispersion in comparison to solid nanoparticles. The spherical rubber particles (0.5–1 μm in diameter) are already predispersed in the commercial product, which helps achieve a relatively uniform distribution.

**Figure 23:**
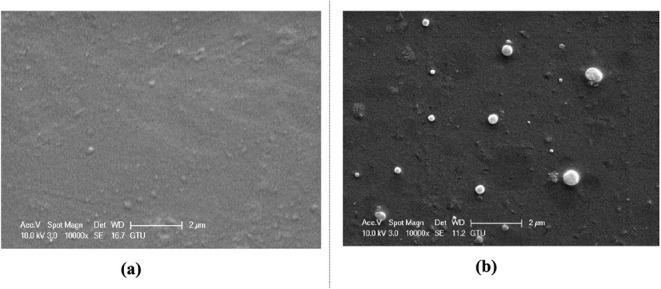
SEM images of different mass fractions of rubber-doped epoxy/rubber samples: (a) unreinforced epoxy and (b) 5% rubber.

#### 4.2.3 HNT- and Rubber-Reinforced Epoxy Samples

The mechanical properties, particularly stiffness and strength, of HNT-doped composites are better/higher than those of rubber-doped composites, primarily due to the stiffness of the reinforcements but also due to the better dispersion of HNT in the epoxy matrix. HNT affects the viscosity significantly less than rubber particles. Particle size, mass fractions, and material nature have significant impacts on this effect on viscosity. As seen from the images, the particle sizes are larger on the surfaces of the rubber-reinforced samples.

Figure 24 shows SEM images of rubber and HNT-reinforced samples. The surfaces of all rubber and HNT-reinforced samples are more heterogeneous and rougher than pure epoxy. The surface structure of 5% rubber and 0.5% HNT-doped epoxy is more heterogeneous than that of the 5% rubber-added and 0.5% HNT-added samples individually. This may be related to the fact that the synergistic effect of both reinforcing materials increases the viscosity, which makes dispersion in the epoxy more difficult than with the materials alone. Similar explanations are also valid for 5% rubber and 1% HNT-reinforced composite. The surface of the 5% rubber and 1% HNT-added epoxy material is more heterogeneous than the epoxy material with 5% rubber and 1% HNT separately. When the amount of HNT in 5% rubber-added epoxy is increased from 0.5% to 1% by weight, it is clearly seen from the SEM images that the number of HNT particles on the material surface increases. On the other hand, when the HNT content in the 5% rubber-added epoxy sample is increased from 0.5% to 1% by weight, some particles are more visible on the surface. Similarly, the surface structure of the 10% rubber and 0.5% HNT-added epoxy sample is more heterogeneous than that of the 10% rubber and 0.5% HNT-added material separately.

**Figure 24:**
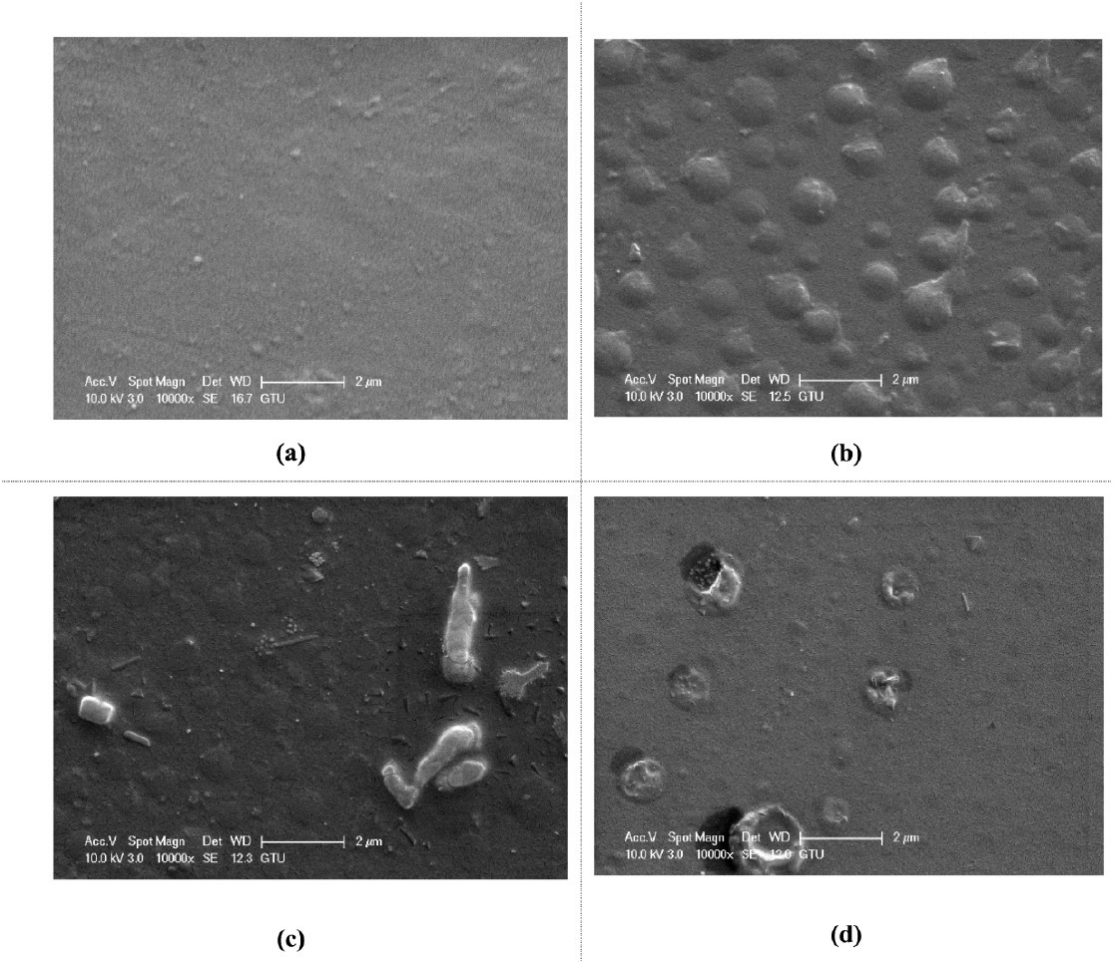
SEM images of different amounts of rubber and HNT-reinforced epoxy samples: (a) unreinforced epoxy, (b) 5% rubber and 0.5% HNT, (c) 5% rubber and 1% HNT, and (d) 10% rubber and 0.5% HNT.

#### 4.2.4 Microstructure–Property Relationships

The observed mechanical property variations correlate directly with the microstructural features revealed by SEM analysis. For HNT reinforcement, well-dispersed nanotubes (Figure 22b) enable effective load transfer from the epoxy matrix to the rigid HNTs (∼140 GPa), thereby increasing stiffness. The high aspect ratio further contributes to crack bridging, modestly enhancing the fracture energy.

For CTBN-modified samples (Figure 23), strong interfacial bonding—achieved through a chemical reaction between carboxyl-terminated CTBN and epoxy groups during curing—is critical for toughening. Under loading, rubber particles undergo cavitation, relieving triaxial stress and promoting matrix shear yielding. This energy-dissipative mechanism substantially enhances toughness at the cost of reduced stiffness due to the rubber’s low modulus (∼5 MPa vs 600 MPa for epoxy). SEM fracture surfaces (Figures 18c, 19c, 21c) show no interfacial debonding or particle pull-out, confirming adequate interfacial strength from covalent bonding.

Hybrid systems (Figure 24) exhibit both mechanisms operating independently without antagonistic interactions. In summary: HNTs increase stiffness via load transfer and crack bridging; CTBN enhances toughness through cavitation-induced shear yielding; the combined system delivers predictable intermediate behavior.

### 4.3 Numerical Modeling

The elasticity modulus values calculated for various strain rates and reinforcements for the composites using the Mori–Tanaka homogenization method are presented in Table 9. As observed in the table, the addition of HNT generally increases the elasticity modulus of the epoxy composite, whereas the rubber additive tends to decrease it. When rubber and HNT are combined as reinforcements, increases and decreases are observed, depending on the mass fractions.

**Table 9:** Elasticity Modules Obtained by the Mori–Tanaka Homogenization Method

	PE	H05	H1	R5	R10	H05R5	H05R10	H1R5	H1R10
Static	2410.8	2614.8	2819.8	2232.3	2069.6	2435.7	2272.5	2640.2	2476.3
5%	2627.7	2833.8	3040.9	2426.2	2243	2631.5	2447.6	2837.7	2653.2
10%	2807.0	3014.4	3222.9	2586.4	2386.1	2793.1	2592.1	3000.7	2799.2

The results obtained using the Halpin–Tsai approach are listed in Table 10. These findings indicate an increase in the modulus of elasticity when HNT is added to the epoxy matrix, while a decrease is observed upon adding rubber. When rubber and HNT reinforcements are used together, increases and decreases are observed, contingent on the mass fractions.

**Table 10:** Elasticity Modulus Obtained by the Halpin–Tsai Model

	PE	H05	H1	R5	R10	H05R5	H05R10	H1R5	H1R10
Static	2410.8	2743.2	3041.5	1784.9	1061.7	2100.0	1336.2	2373.9	1559.7
5%	2627.7	2964.6	3269	1921.7	1101.4	2241.5	1379.1	2521.8	1606.4
10%	2807.0	3146.9	3455.7	2033.7	1131.8	2357.0	1411.9	2642.0	1642.0

In their study, Sheng et al. compared the Halpin–Tsai and Mori–Tanaka models, noting that the values obtained in both models display similar trends, with the Halpin–Tsai model producing somewhat more rigid results and acceptable data. A review in the literature reveals that the Mori–Tanaka model is most effective for large aspect ratio reinforcements.^88^ Another study found that the Halpin–Tsai model yields reasonable results with low-weight reinforcement content.^89^ Consequently, the Halpin–Tsai model did not offer consistent results at higher mass fraction cases but still provided predictability for material behavior.

The results obtained from the FEH approach are presented in Table 11. From the results, it can be clearly said that the FEH approach results are consistent with other numerical method results and experimental data. In addition, different from other approaches, the stress distribution over the RVE is obtained with this approach. In HNT-reinforced cases, since HNT reinforcement enhances the composite materials’ elasticity modulus, maximum stresses occur around the reinforcement particles. Quite the opposite, since rubber reinforcement has a lower elasticity modulus than epoxy, maximum stresses occur in the matrix cases. Since tensile and flexural tests are conducted, the maximum stress criteria are evaluated in two cases: Von Mises and Tresca. Results for rubber-reinforced samples are given in Figures 25, 26, and 27 . Additionally, reinforcement leads to irregularity and heterogeneity in the composite; failure of the composite structure will be observed there.

**Figure 25:**
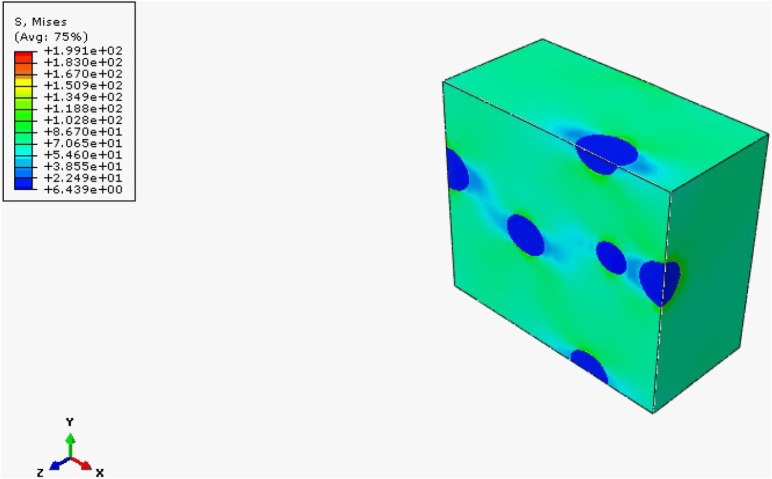
Von Mises stress distribution of mf = 5% rubber reinforced composite.

**Figure 26:**
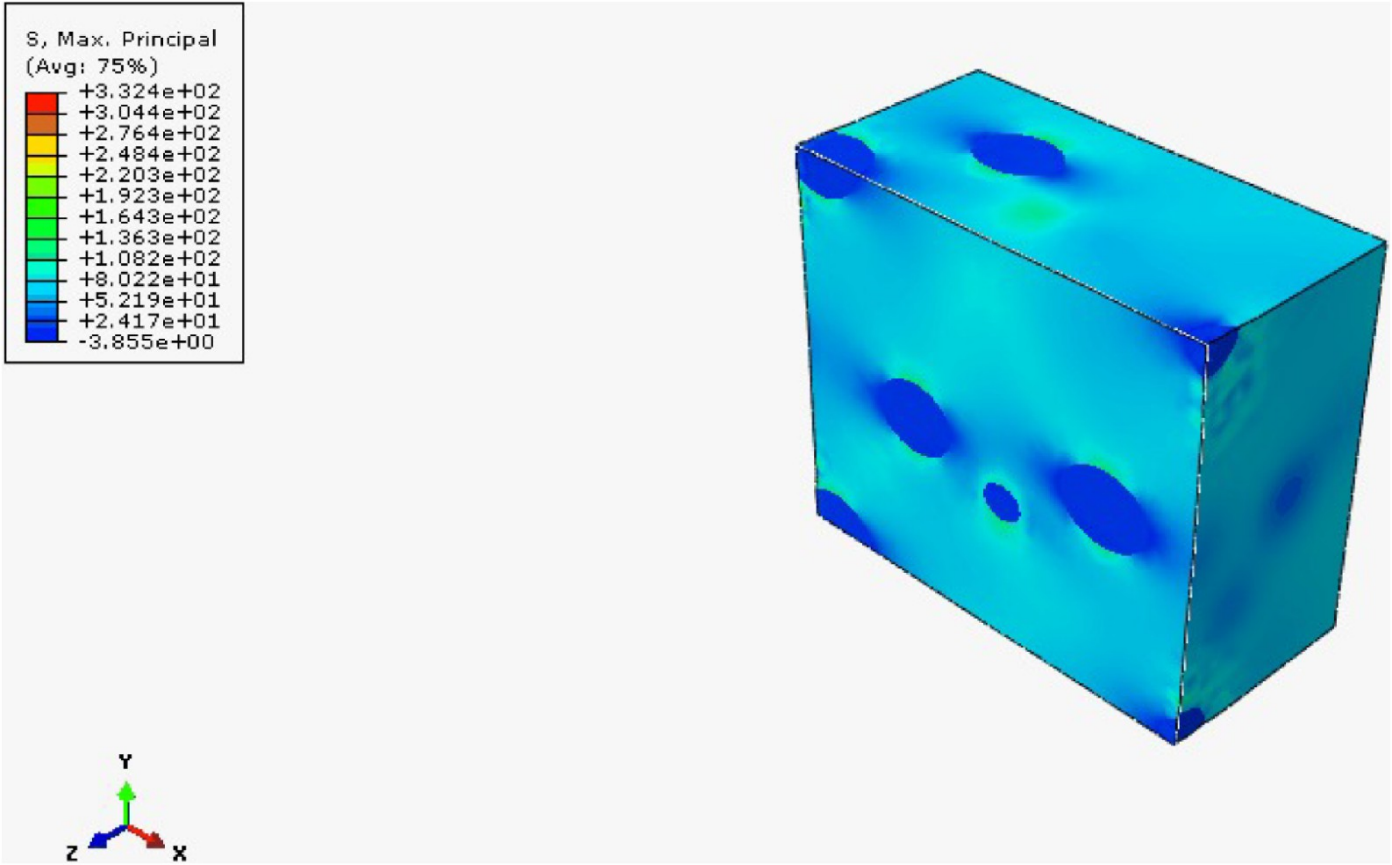
Maximum principal stress distribution of mf = 10% rubber-reinforced composite.

**Figure 27:**
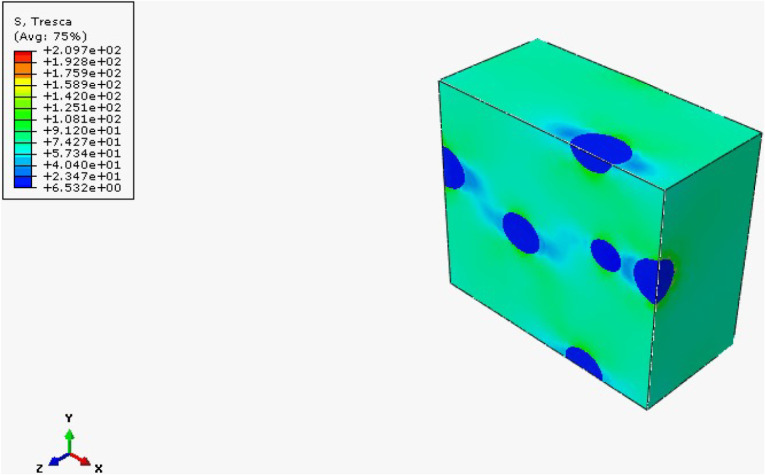
Tresca stress distribution of mf = 5% rubber-reinforced composite.

**Table 11:** Elasticity Modulus Obtained by the FEH Approach

	PE	H05	H1	R5	R10	H05R5	H05R10	H1R5	H1R10
Static	2410.8	2690.4	2792.3	2309.7	2105.3	2476.9	2254.6	2714.9	2464.9
5%	2627.7	2787.2	3026.0	2534.3	2345.2	2607.7	2480.2	2884.5	2687.2
10%	2807.0	2995.1	3203.8	2602.3	2508.2	2712.0	2523.6	2988.0	2801.7

Comparing the numerical modeling results with the literature, it becomes apparent that adding HNT to the epoxy matrix enhances the modulus of elasticity of the epoxy composite, while adding rubber reduces it. It can be stated that similar findings have also been reported in other studies,.^15,90^

In order to compare results visually, comparison plots for a single reinforcement and a bar graph for a synergistic effect are given in Figures 28, and 29,30. It is seen from these figures that experimental and numerical results have consistency with each other. The FEH approach has the most accuracy, and Halphin–Tsai is the least accurate one.

**Figure 28:**
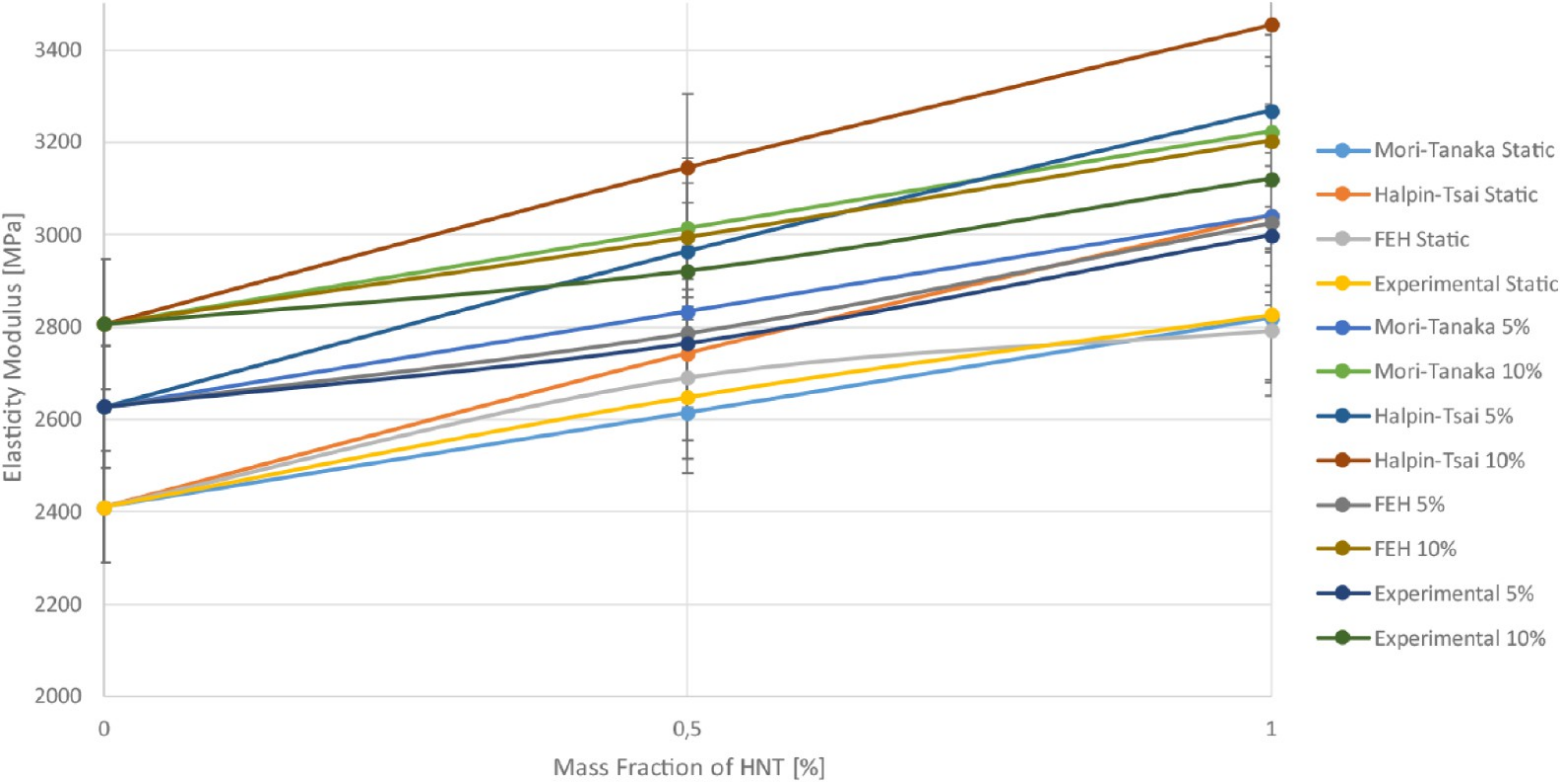
Comparison of numerical and experimental results of the HNT reinforcement case.

**Figure 29:**
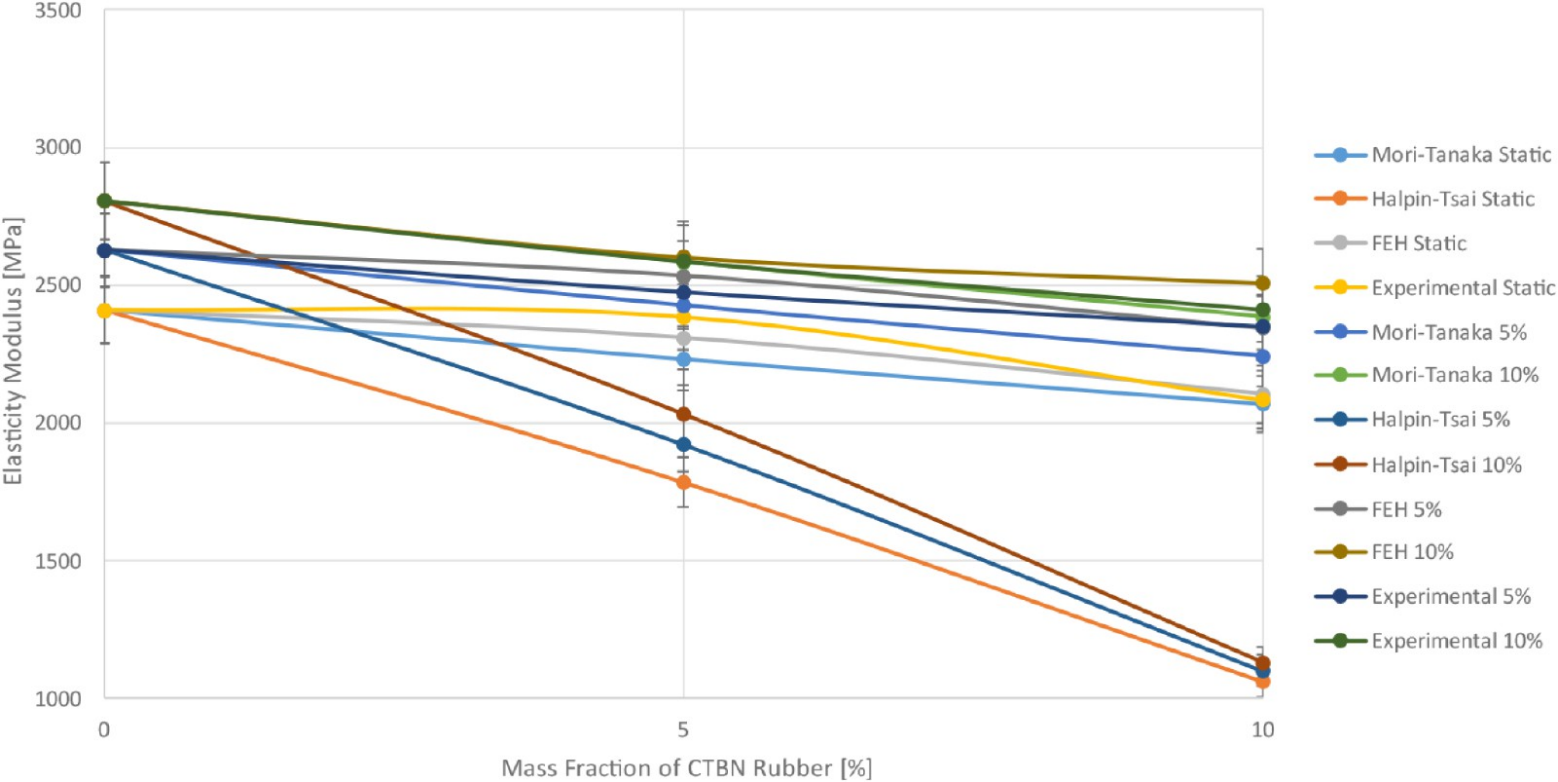
Comparison of numerical and experimental results of the CTBN rubber-reinforced case.

**Figure 30:**
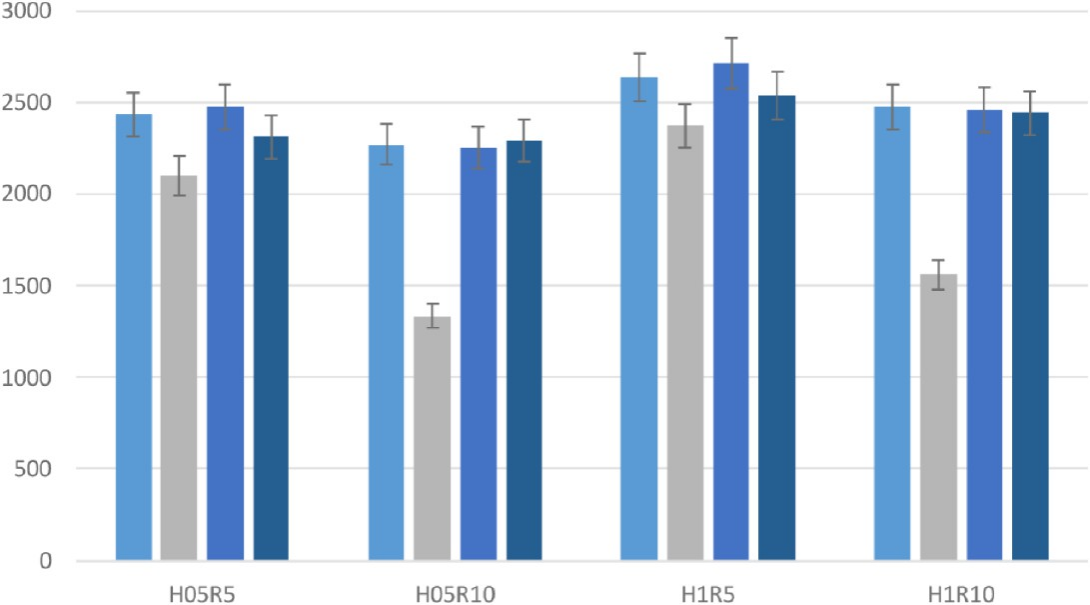
Comparison of numerical and experimental results of the synergetic effect case.

## 5 Conclusions

This study investigates epoxy-based nanocomposites reinforced with halloysite nanotubes (HNT), carboxyl-terminated butadiene-acrylonitrile (CTBN) rubber, and their combinations, focusing on their mechanical characterization as a function of strain rate and impact performance. To this end, experimental and numerical analyses are conducted, and the results are presented. A standardized manufacturing procedure ensures consistency across all samples, with pure epoxy serving as the control group. Following the manufacturing process, the samples undergo experimental procedures such as tensile tests, three-point bending tests, Charpy impact tests, and SEM.

Tensile test results reveal that HNT reinforcement increases stiffness and strength but reduces ductility, while CTBN rubber enhances ductility at the expense of rigidity. HNT reinforcement increases composite stiffness: 1% HNT provides 15% higher tensile elastic modulus (688 vs 599 MPa) and 17% higher flexural modulus (2827 vs 2411 MPa) compared to pure epoxy at quasi-static rates. CTBN rubber addition significantly enhances ductility: 10% rubber increases elongation at break by 48% (0.142 vs 0.096) but reduces tensile stiffness by 38% (370 vs 599 MPa). The hybrid sample H10R05 exhibits intermediate properties: an elasticity modulus of 503 MPa (16% higher than R10 alone) while maintaining elongation at break of 0.138 (44% higher than pure epoxy), demonstrating predictable property balancing. All samples exhibit strain-rate-dependent behavior, with stiffness increasing at higher strain rates, characteristic of the epoxy matrix’s viscoelastic nature. Elasticity modulus increases consistently with strain rate across all samples: an average 10–16% increase from 0.01 to 0.1 strain/min, confirming that the viscoelastic nature of the epoxy matrix dominates the overall composite response. Upon examination of the three-point bending test results, it is evident that all samples exhibit greater rigidity and higher elongation at break than in the tensile test results. This highlights that the material’s behavior is dependent on the direction and type of applied load. Moreover, the three-point bending tests generally show increased rigidity at higher strain rates. In terms of reinforcement material effects, similar to the tensile test results, stiffness increases with the HNT additive, while rubber addition enhances the structure’s damping properties. The differences between the flexural and tensile test results highlight the importance of load direction and stress distribution in assessing material behavior.

Charpy impact test results demonstrate that the fracture properties of the composite are improved with the addition of HNT and rubber. The highest fracture energy is observed in the 10% rubber-reinforced samples. Combinations of HNT and rubber yield fracture properties different from those of single-reinforcement systems, indicating no unexpected interactions. Charpy impact tests reveal that R10 provides the highest fracture energy (85% higher than that of pure epoxy), while H10 shows modest improvements (12% higher). Hybrid systems exhibit intermediate values, confirming additive rather than synergistic fracture behavior.

The Mori–Tanaka homogenization method, Halpin–Tsai model, and finite element homogenization (FEH) approach successfully predict experimental trends. The FEH approach provides additional insights through stress distribution analysis, identifying maximum stress locations around HNT particles and within the matrix for rubber-reinforced composites. While requiring higher computational costs, FEH offers superior accuracy and detailed mechanical insights compared to analytical methods. The FEH approach shows the highest accuracy (deviations <5% from experimental values), while Mori–Tanaka provides reasonable predictions (deviations ∼8–12%) and Halpin–Tsai shows larger deviations (15–40%), particularly for rubber-rich compositions.

In conclusion, employing multiple reinforcement types with differing mechanical behaviors can be advantageous for designing composite materials with balanced stiffness, ductility, and toughness. This study demonstrates that multireinforcement strategies enable the tailoring of mechanical properties in epoxy composites. The predictable synergistic behavior of HNT and CTBN rubber allows for design optimization, balancing stiffness, strength, and toughness. The mass fractions investigated in this study (0.5–1% HNT and 5–10% CTBN) were selected based on manufacturing feasibility and literature precedent. Higher reinforcement contents were not explored due to anticipated challenges, including increased viscosity, potential agglomeration (particularly for HNT), and excessive stiffness reduction (for CTBN). Future studies employing advanced dispersion techniques, such as high-shear mixing, three-roll milling, or solvent-assisted processing, could explore higher loading levels to identify optimal reinforcement contents for specific applications. Understanding the upper limits of reinforcement addition and the transition from beneficial to detrimental effects remains an important area for continued research. In the future, examining the design space for advanced composite materials by varying mass fractions and alternative reinforcements will be crucial for designing composite materials and broadening their application areas. Investigation of long-term durability and environmental effects would further enhance the applicability of these nanocomposites in engineering applications.

## Data Availability

No data are generated during this research.
